# AGAPIR: A Novel PIWI‐Interacting RNA Enhancing Post‐Decompression Angiogenesis in Degenerative Cervical Myelopathy

**DOI:** 10.1002/advs.202504246

**Published:** 2025-08-18

**Authors:** Yongheng Xie, Yiling Peng, Tianyu Qin, Cuimei Chen, Zhenxiao Ren, Naibo Feng, Chungeng Liu, Songlin Peng, Houqing Long

**Affiliations:** ^1^ Division of Spine Department of Orthopedic Surgery Shenzhen People's Hospital (The First Affiliated Hospital Southern University of Science and Technology The Second Clinical Medical College Jinan University) Shenzhen 518020 China; ^2^ The First Affiliated Hospital Jinan University Guangzhou 510630 China; ^3^ Shenzhen Key Laboratory of Musculoskeletal Tissue Reconstruction and Function Restoration Shenzhen 518020 China; ^4^ Guangdong Provincial Key Laboratory of Orthopaedics and Traumatology/Department of Spine Surgery The First Affiliated Hospital Sun Yat‐sen University Guangzhou 510000 China; ^5^ Department of Cardiovascular Surgery Guangdong Cardiovascular Institute Guangdong Provincial People's Hospital Guangdong Academy of Medical Sciences Southern Medical University Guangzhou 510000 China; ^6^ Guangdong Provincial Clinical Research Centre for Geriatrics Shenzhen Clinical Research Centre for Geriatrics Department of Geriatrics Shenzhen People's Hospital Shenzhen 518020 China

**Keywords:** angiogenesis, degenerative cervical myelopathy, HIF‐1α, PIWI‐interacting RNA, USP18

## Abstract

Degenerative cervical myelopathy (DCM) is the most common cause of spinal cord dysfunction worldwide. Although surgical decompression can halt disease progression and improve neurological function in most patients, there remains a subset for whom functional improvement is limited. Impaired spinal cord perfusion is a pathological hallmark of DCM, which highlights the importance of restoring blood flow to enhance neurological outcomes. Here, this work identifies a novel angiogenesis‐associated PIWI‐interacting RNA (AGAPIR) that enhances angiogenesis and motor function following spinal cord decompression. Using piRNA sequencing, this work identifies AGAPIR as a key regulator of DCM pathogenesis. This work observes significant upregulation of AGAPIR expression in the spinal cord following surgical decompression, which is correlated with enhanced angiogenesis. Overexpression of AGAPIR markedly improves blood vessel formation and promotes motor function in mice following spinal cord decompression. Using RNA sequencing and cellular validation, this work finds that AGAPIR directly interacts with USP18, leading to an increase in its protein expression level and stabilization of HIF‐1α protein via its deubiquitinating activity. This data demonstrate that AGAPIR is a novel piRNA that further improves the functional status of DCM mice following surgical decompression. These results provide the pioneering evidence and novel insights into piRNA‐directed therapeutic strategies for DCM.

## Graphical abstract

The restoration of blood flow following surgical decompression for degenerative cervical myelopathy (DCM) significantly contributes to the amelioration of neurological deficits. This study identifies AGAPIR, an angiogenesis‐associated PIWI‐interacting RNA, enhances angiogenesis and motor function recovery post‐spinal cord decompression in a mouse model of DCM. Mechanistically, AGAPIR facilitates angiogenesis through the modulation of the USP18/HIF‐1α axis.

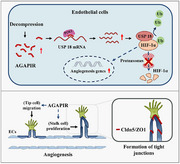

## 1 Introduction

DCM is the leading cause of spinal cord dysfunction among adults worldwide and encompasses a broad range of degenerative arthritic and congenital conditions of the cervical spine, including cervical spondylosis and ossification of the posterior longitudinal ligament. Its prevalence is increasing rapidly, threatening 22% of individuals aged ≥ 65 y in the United States by 2050.^[^ ^1^ ^]^ Currently, surgical decompression is the most recommended treatment for patients with moderate‐to‐severe DCM.^[^ ^2^ ^]^ However, given the individual differences in the symptoms of this disease in the early stages, most patients may not seek prompt medical assistance until severe neurological impairment has developed, which substantially diminishes the effectiveness of surgical intervention.^[^ ^1^, ^3^ ^]^ Therefore, some patients experience varying degrees of neurological dysfunction after surgical decompression.^[^ ^4^ ^]^ Moreover, no effective pharmacological agents have been shown to restore the neurological function after surgical decompression.^[^ ^5^ ^]^ Impaired spinal cord perfusion has long been considered the central pathophysiological tenet of DCM.^[^ ^1^ ^]^ Chronic compression leads to spinal cord ischemia, resulting in chronic inflammation and neuronal apoptosis.^[^ ^1^ ^]^ Thus, effective strategies to restore spinal cord perfusion may further improve neurological function in patients with DCM who have undergone decompression surgery.

Recently, increasing evidence has indicated that non‐coding RNAs play a significant role in the pathogenesis of neurological diseases, including DCM.^[^ ^6^, ^7^ ^]^ Among the various types of non‐coding RNAs, PIWI‐interacting RNAs (piRNAs), characterized by a strong uracil bias at the 5ʹ‐terminal and 2ʹ‐O‐methylation at the 3ʹ‐terminal, with lengths ranging from 26 to 35 nucleotides, were initially identified as a novel class of small non‐coding RNAs in the mammalian germline.^[^ ^8^, ^9^, ^10^, ^11^ ^]^ In recent years, piRNA has been widely identified in diverse mammalian tissues and organs beyond the reproductive system and has demonstrated essential regulatory functions. These encompass the cardiovascular system (especially endothelial cells),^[^ ^12^, ^13^, ^14^, ^15^, ^16^, ^17^ ^]^ and multiple organ systems, including the lungs,^[^ ^18^, ^19^ ^]^ liver,^[^ ^20^ ^]^ kidneys,^[^ ^21^ ^]^ breast,^[^ ^22^ ^]^ and skeletal muscles.^[^ ^23^ ^]^ Additionally, piRNAs are widely expressed in the nervous system and are involved in modulating various neurophysiological and neuropathological processes.^[^ ^24^, ^25^ ^]^ An angiogenesis‐associated piRNA, piR‐1245, was recently reported to promote retinal neovascularization.^[^ ^26^ ^]^ piRNAs are emerging as crucial regulators of endothelial cell proliferation and play a significant role in angiogenesis.^[^ ^22^, ^27^ ^]^ Nonetheless, our knowledge of whether piRNAs modulate spinal cord angiogenesis following surgical decompression remains limited, and the mechanisms by which they regulate endothelial cell function remain poorly understood.

In the normal spinal cord, endothelial cells remain in a quiescent state.^[^ ^28^ ^]^ However, after sustained spinal cord injury, quiescent endothelial cells undergo rapid migration and proliferation during angiogenesis. Chronic compression of the spinal cord microvasculature leads to hypoxia, which triggers activation of spinal cord microvascular endothelial cells (SCMECs).^[^ ^1^ ^]^ HIF‐1α is a major determinant of angiogenesis by inducing the transcription of vascular endothelial growth factor (VEGF).^[^ ^29^ ^]^ The stability of HIF‐1α protein is regulated by ubiquitination and proteasome‐dependent degradation, and this process can be antagonized by deubiquitinating enzymes (DUBs).^[^ ^30^ ^]^ The mammalian genome encodes ≈100 DUBs, and the largest subclass of DUBs is the ubiquitin‐specific proteases (USPs). A previous report demonstrated the significant role of USPs in the modulation of HIF‐1α.^[^ ^31^, ^32^ ^]^ However, the exact roles of USPs and their regulatory mechanisms in ischemia‐induced angiogenesis in DCM remain unknown.

In this study, we aimed to investigate the functional piRNAs implicated in DCM pathogenesis and elucidate the underlying mechanisms involved in angiogenesis. Our findings indicated that angiogenesis‐associated piRNA (AGAPIR) was significantly upregulated, accompanied by a notable increase in angiogenesis in the spinal cord tissue from surgically decompressed DCM mice. Subsequent investigations demonstrated that AGAPIR promoted angiogenesis both in vitro and in vivo. Furthermore, AGAPIR has been shown to stabilize the HIF‐1α protein through its interaction with USP18, a deubiquitinating enzyme. Thus, AGAPIR overexpression may serve as a promising novel therapeutic approach to enhance neurological recovery following spinal cord decompression.

## 2 Results

### 2.1 Surgical Decompression Enhances Angiogenesis and Motor Recovery in DCM Mice

**Figure 1:**
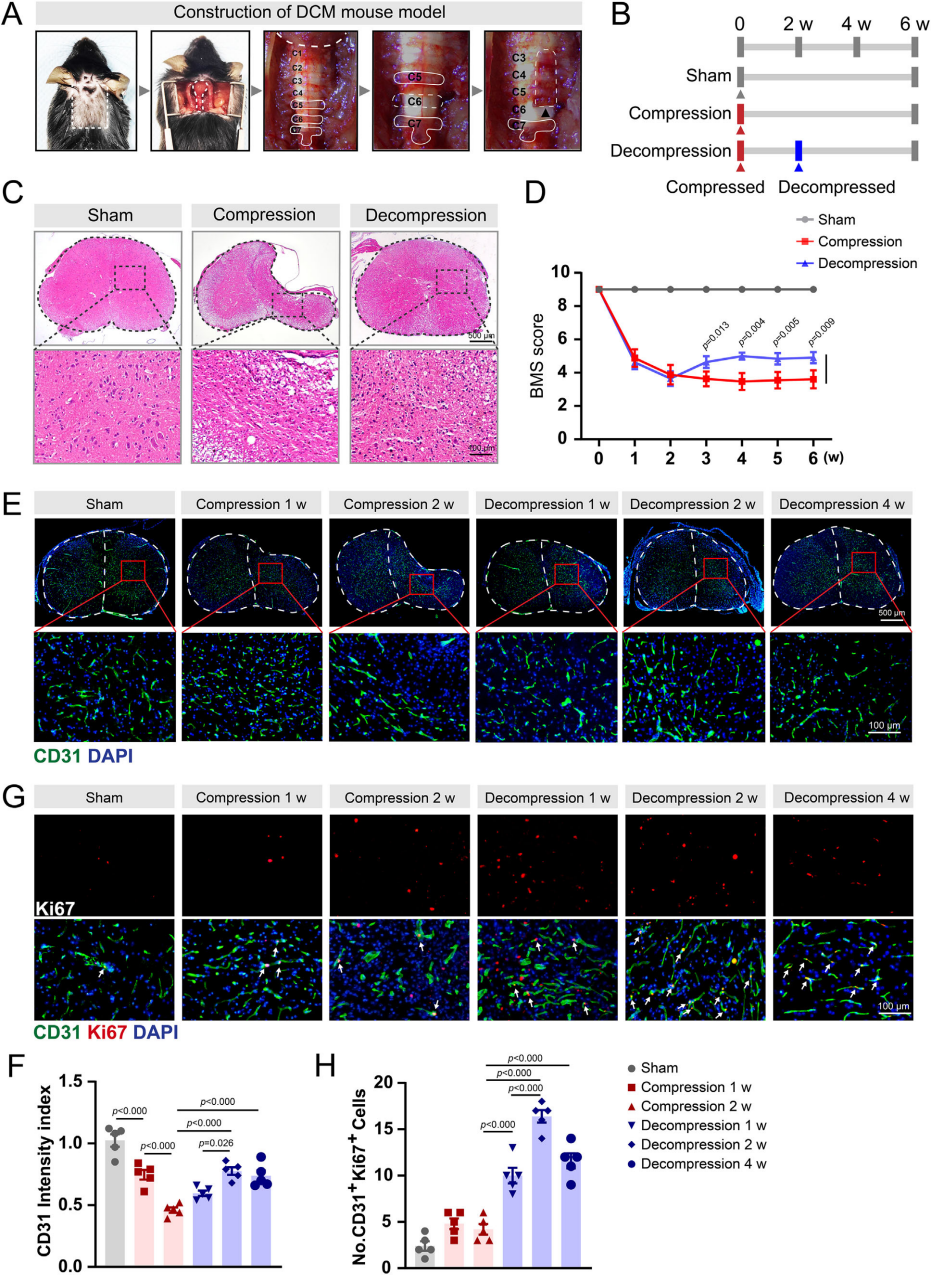
Surgical decompression enhances spinal cord vascularization and functional recovery in a chronic cervical compression model. A) Schematic representation of the chronic cervical spinal cord compression injury model in mice. B) Experimental timeline outlining surgical interventions and postoperative assessments across groups. C) Histopathological evaluation by hematoxylin & eosin (H&E) staining of cervical spinal cord sections from sham‐operated controls, 6‐week compression, and 4‐week post‐decompression groups. D) Longitudinal assessment of motor function using Basso Mouse Scale (BMS) scores at weekly intervals (1–6 weeks post‐surgery). E) Representative immunohistochemical staining and F) quantitative analysis of CD31⁺ vascular density (mean fluorescence intensity = integrated density / region of interest area) in lesioned versus adjacent intact spinal cord regions. G) Dual immunofluorescence detection of proliferating endothelial cells (CD31⁺, green; Ki67⁺, red) and H) quantification of CD31⁺Ki67⁺ double‐positive cells per mm^2^. Data represent mean ± SEM (n = 5/group); Statistical significance was determined by one‐way ANOVA with Bonferroni's (D, F, H) or Dunnett's T3 (D) multiple comparison test.

Impaired spinal cord perfusion is recognized as a central pathophysiological tenet in DCM;^[^ ^1^ ^]^ making restoration of vascular perfusion after decompression critical for neurological recovery.^[^ ^33^ ^]^ To investigate the vascular remodeling dynamics, we generated a chronic spinal cord compression model in mice using hydrophilic polymer sheets, following established protocols (**Figures** **1**A and S1A, Supporting Information). Our previous observations demonstrated that animals subjected to chronic spinal cord compression exhibited a progressive decline in motor function, which reached its lowest point at 2 weeks post‐compression. However, despite the prolonged duration of compression, no further significant deterioration in spinal cord dysfunction was observed. Based on these findings, surgical decompression was performed at 2 weeks post‐implantation, followed by euthanasia of the mice 4 weeks after decompression (Figure 1B). Quantitative morphometric analysis revealed significant spinal cord volume loss under compression (Figure 1C), which was nearly fully restored at 4 weeks post‐decompression, concomitant with an increase in the total cell number. We then assessed motor function using Basso Mouse Scale (BMS) scores, which showed a significant drop at 2 weeks post‐compression and remained low for four weeks. In the decompression group, the BMS scores improved progressively, peaking at 2 weeks post‐surgery (Figure 1D).

To evaluate angiogenic activity, immunofluorescence staining for CD31 (a marker for endothelial cells) and α‐SMA (a marker for smooth muscle cells in blood vessels) was conducted on spinal cord sections. Chronic spinal cord compression led to a gradual reduction in the number of CD31‐positive and α‐SMA‐positive blood vessel cells (Figure 1E,F, Figure S1B,C, Supporting Information). Conversely, surgical decompression partially restored vascular density, resulting in an increase in the number of CD31‐positive and α‐SMA‐positive cells, which peaked 2 weeks after decompression. These dynamic changes in endothelial cells parallel the timeline of recovery of neurological function during compression and decompression. Furthermore, we investigated endothelial cell proliferation using double immunofluorescence staining for Ki67 (red) and CD31 (green) in spinal cord sections. Immunofluorescence results indicated that chronic compression increased the percentage of Ki67 and CD31 double‐positive cells compared with the sham group. Notably, surgical decompression significantly elevated the number of Ki67/CD31 double‐positive cells compared with the compression group, with peak numbers observed 2 weeks after decompression. A substantial number of proliferating CD31‐positive cells persisted even 4 weeks after decompression (Figure 1G,H), indicating that endothelial cell proliferation occurred during compression and was significantly enhanced following surgical decompression.

Single‐cell RNA sequencing (scRNA‐seq) of chronically compressed rat cervical spinal cords revealed transcriptomic shifts toward angiogenic patterns in endothelial cells. From 5796–8998 high‐quality single‐cell transcriptomes per sample (n = 3/group), we identified 13 major cell classes, including oligodendrocytes, microglia, and endothelial cells (Figure S2A,B, Supporting Information). Five subtypes of endothelial cells were identified, showing significant differences in proportions between the groups (Figure S2C, Supporting Information). Specifically, subtypes 0 and 4 increased, whereas subtypes 1, 2, and 3 decreased in the compression group (Figure S2D, Supporting Information). Subtype 0 gene expression profiles were associated with angiogenesis, including *Flt1*, *Slco1c1*, *Ptprb*, *Sptbn1*, and *Syne1* (Figure S2E, Supporting Information), and Gene Ontology (GO) term analysis revealed enrichment in angiogenesis‐related biological processes (Figure S2F, Supporting Information). Additionally, the gene expression profiles in the compression group suggested the activation of tip cells and angiogenesis (Figure S2G,H, Supporting Information). Together, these findings indicate that chronic spinal cord compression induces both a reduction in endothelial cell numbers and compensatory angiogenesis, and that surgical decompression sustains enhancement of this endogenous repair mechanism.

### 2.2 Identification and Characterization of Angiogenesis‐Associated piRNAs

**Figure 2:**
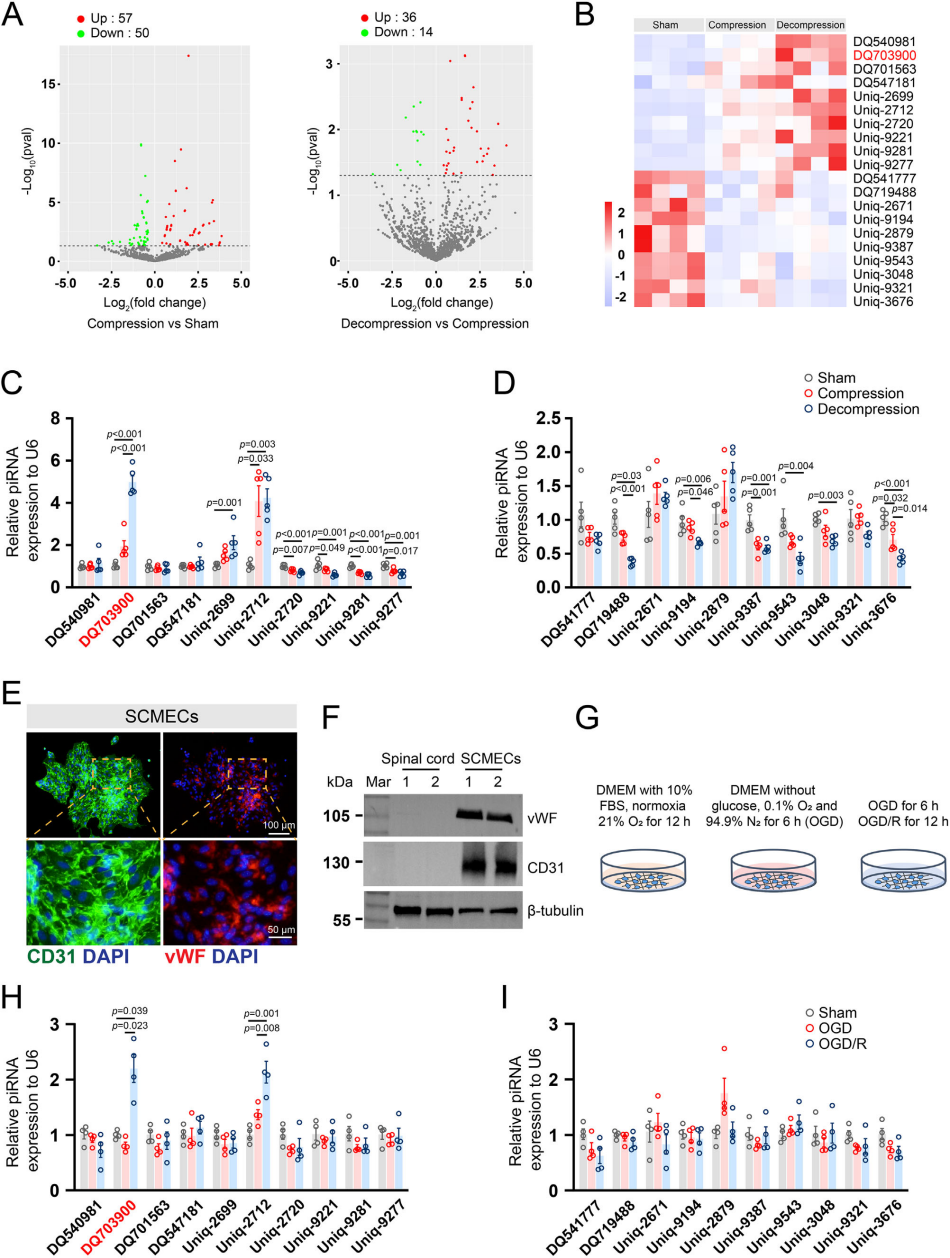
Identification of angiogenesis‐associated piRNAs in spinal cord vascular remodeling. A) Volcano plot illustrating differentially expressed piRNAs across experimental groups (Compression versus sham, Decompression versus Compression; |log_2_FC| >1, *p* < 0.05). B) The heat map shows the top 10 most significantly upregulated (red) and downregulated (blue) piRNAs (n = 5/group). C,D) Quantitative real‐time polymerase chain reaction validation of candidate piRNAs (top 10 upregulated/downregulated) in spinal cord tissues (n = 5/group). E) Immunofluorescence colocalization of endothelial markers CD31 (green) and von Willebrand Factor (vWF, red) in primary murine spinal cord microvascular endothelial cells (SCMECs). F) Western blot analysis confirming CD31 and vWF protein expression in SCMECs (β‐tubulin loading control). G) Experimental workflow for SCMEC treatments: normoxia (21% O_2_), oxygen‐glucose deprivation (OGD, 0.1% O_2_), and OGD/reperfusion (OGD/R). H,I) Expression profiles of mechanistically relevant piRNAs in SCMECs under normoxic, OGD, and OGD/R conditions (n = 4/group). Data represent mean ± SEM; Statistical significance was determined by one‐way ANOVA with Bonferroni's or Dunnett's T3 multiple comparison test.

To characterize the piRNA profiles in the cervical spinal cord, we conducted piRNA sequencing of tissues from sham‐operated, compressed, and decompressed mice (n = 4/group). Our analysis identified 107 differentially expressed piRNAs in the compression group, with 57 upregulated and 50 downregulated piRNAs compared to those in the sham group. Additionally, we found 50 differentially expressed piRNAs in the decompression group, including 36 upregulated and 14 downregulated piRNAs, compared to those in the compression group (**Figure** **2**A). To explore whether piRNAs regulate endothelial cell proliferation, we identified the top 20 dysregulated piRNAs ranked by fold change, comprising 10 sequentially upregulated and 10 sequentially downregulated piRNAs across the sham, compression, and decompression groups, using a heatmap (Figure 2B). The trends of piRNA upregulation and downregulation were consistent with the proliferative trends observed in endothelial cells across these groups. Subsequently, RT‐qPCR was conducted to validate the expression levels of these piRNAs in cervical spinal cord tissues from sham, compression, and decompression mice. Among the validated top 10 upregulated piRNAs, three demonstrated increased expression, with DQ703900 (mus‐piR‐119222) showing the most significant upregulation (Figure 2C). Among the top ten downregulated piRNAs, six exhibited marked decreases, with DQ719488 (mus‐piR‐134810) being the most notable (Figure 2D).

We isolated and cultured SCMECs in vitro following established protocols.^[^ ^34^ ^]^ Immunofluorescence staining confirmed that nearly all cells expressed the endothelial cell surface markers CD31 and von Willebrand Factor (vWF) (Figure 2E), which was further validated by western blot analysis (Figure 2F). To recapitulate the in vivo microenvironment, we established an oxygen‐glucose deprivation/reoxygenation (OGD/R) model: 12 h normoxia (sham), 6 h OGD (compression), and 6 h OGD +12 h reoxygenation (decompression) (Figure 2G). We collected cells and conducted RT‐qPCR to validate the expression of the aforementioned piRNAs. Notably, the expression level of DQ703900 significantly increased in the OGD/R‐treated group. These results suggest that the upregulation of DQ703900 in decompression conditions may be involved in SCMECs angiogenesis. Consequently, we termed the DQ703900 angiogenesis‐associated piRNA (AGAPIR).

**Figure 3:**
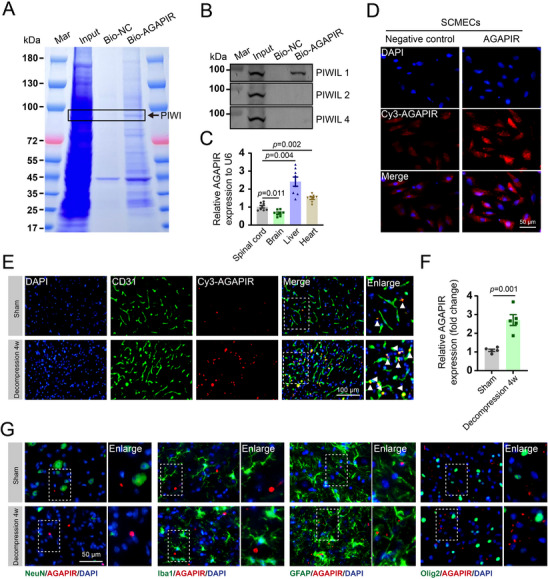
Characterization of angiogenesis‐associated piRNAs. A) RNA pull‐down assay using biotinylated AGAPIR probes or negative control probes. B) Validation of AGAPIR‐PIWIL interaction through biotin‐based pull‐down assays. Proteins bound to Bio‐AGAPIR were analyzed by western blotting for PIWIL1, PIWIL2, and PIWIL4 expression. C) Tissue‐specific expression profiling of AGAPIR by quantitative real‐time polymerase chain reaction across spinal cord, brain, liver, and heart tissues (n = 8/group). Statistical significance was assessed via one‐way ANOVA with Dunnett's T3 post hoc analysis. D) Fluorescence in situ hybridization (FISH) using Cy3‐labeled AGAPIR probes (red) revealed specific expression in spinal cord microvascular endothelial cells (SCMECs). E) Comparative FISH analysis of AGAPIR expression in spinal cord sections from sham‐operated versus decompression‐treated mice. F) Quantification of AGAPIR‐CD31 colocalization signals (n = 5/group). Data were analyzed using two‐tailed Student's *t*‐test. G) FISH coupled with immunofluorescence staining revealed AGAPIR's spatial colocalization with key neural lineage markers in spinal cord tissues: NeuN⁺ neurons, Iba1⁺ microglia, GFAP⁺ astrocytes, and Olig2⁺ oligodendrocytes.

Subsequently, biotin‐streptavidin pull‐down assays using biotinylated AGAPIR in SCMECs were performed to identify binding partners. As shown in **Figure** **3**A, several bands were enriched in the biotinylated AGAPIR pull‐down group. An RNA pull‐down assay followed by immunoblotting confirmed that PIWI‐like protein 1 (PIWIL1), a functional partner of piRNAs, binds to AGAPIR to form a complex in SCMECs (Figure 3B). Furthermore, RT‐qPCR was used to investigate the distribution pattern of AGAPIR across the spinal cord, brain, liver, and heart. The results revealed distinct expression levels of AGAPIR in these tissues. Expression in the brain was relatively low, whereas higher expression was observed in the liver and heart than in the spinal cord (Figure 3C). This indicates that systemic treatment with AGAPIR would require enhanced targeting, specifically to the spinal cord vasculature, to prevent unwanted effects on the liver and heart.

To verify AGAPIR expression in SCMECs both in vivo and in vitro, we conducted fluorescence in situ hybridization (FISH) experiments using Cy3‐labeled AGAPIR and a negative control antisense probe. The results showed that AGAPIR was highly expressed in SCMECs (Figure 3D). Moreover, RNA FISH combined with immunofluorescence staining for cell type‐specific markers demonstrated that AGAPIR (Cy3‐labeled, red) predominantly colocalized with CD31^+^ (green) endothelial cells in both the sham and decompression groups. In the sham group, endothelial cells remained in a quiescent state and exhibited basal AGAPIR expression, whereas in the decompression group, activated endothelial cells showed significantly elevated AGAPIR expression (yellow regions) (Figure 3E,F). This differential expression pattern highlights the potential role of AGAPIR in endothelial cell activation under decompression conditions. We also systematically evaluated AGAPIR expression in other neural cell types, including neurons (NeuN), microglia (Iba1), astrocytes (GFAP), and oligodendrocytes (Olig2). Importantly, AGAPIR showed negligible colocalization (yellow regions) with these markers across experimental conditions, suggesting that its functional role is specific to endothelial cells (Figure 3G). These findings suggest that AGAPIR acts as a critical regulator of angiogenesis in the spinal cord during decompression.

### 2.3 AGAPIR Promotes Angiogenesis In Vitro

**Figure 4:**
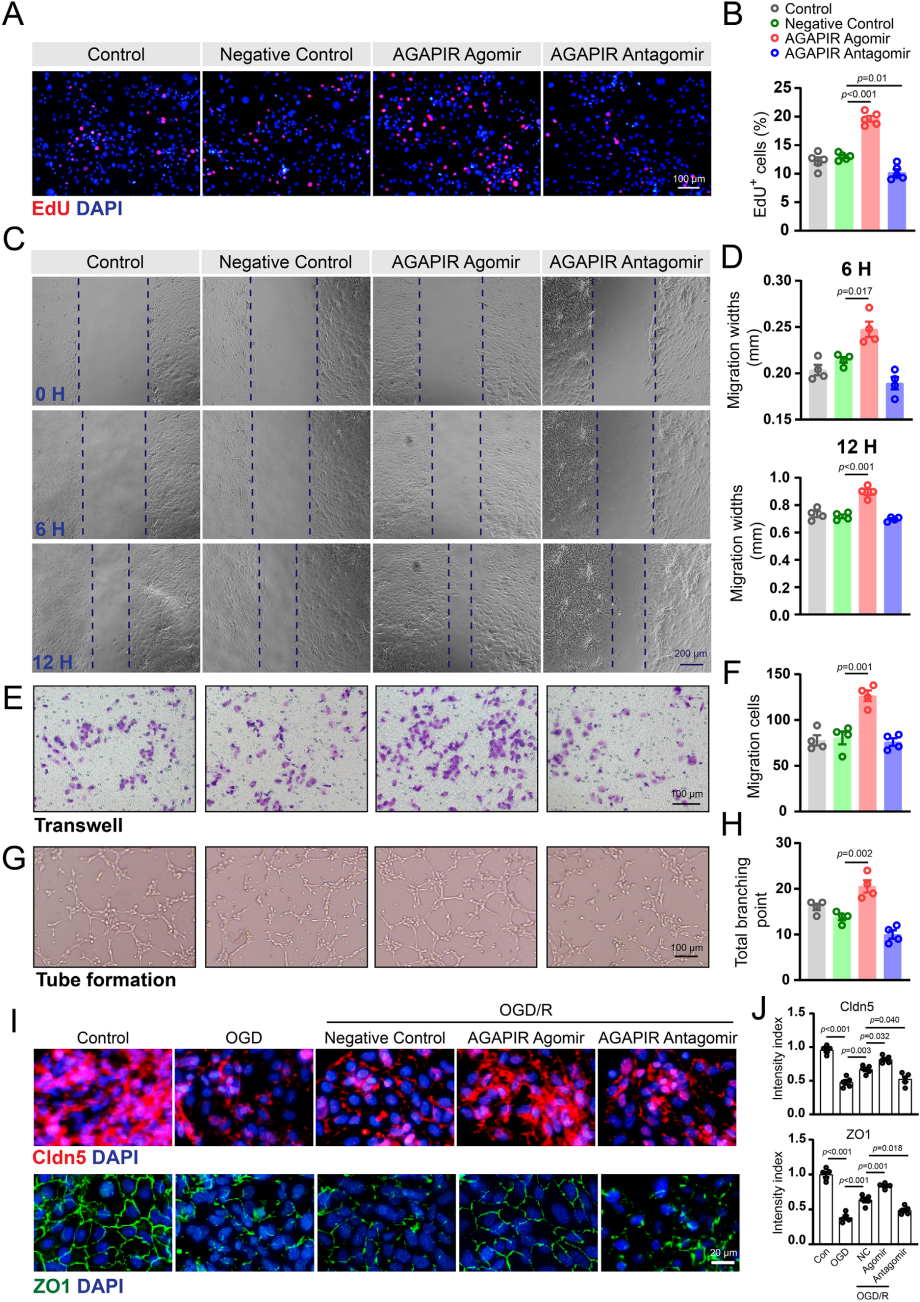
AGAPIR promotes angiogenic activities of SCMECs in vitro. A) Representative immunofluorescence images of EdU incorporation assay and B) quantitative analysis of EdU‐positive cell rates in SCMECs treated with control, negative control, AGAPIR agomir, or AGAPIR antagomir. Data are presented as mean ± SEM; n = 5/group. C) Representative images of scratch wound healing assay in bEnd.3 cells under indicated treatments and D) quantitative analysis of wound closure area. E) Representative images of transwell migration assay and F) corresponding quantification of migrated bEnd.3 cells. G) Representative images of tube formation assay and H) quantitative assessment of total branching points in bEnd.3 cells. I) Immunofluorescence staining of tight junction proteins (Claudin‐5 and ZO‐1) in SCMECs exposed to oxygen‐glucose deprivation (OGD) or OGD/reoxygenation (OGD/R) conditions. J) Quantification of Claudin‐5 and ZO‐1 fluorescence intensity (n = 4/group). Statistical significance was determined by one‐way ANOVA with Bonferroni's multiple comparison test.

To investigate the effects of AGAPIR overexpression and silencing on endothelial cells, we synthesized an AGAPIR agomir and antagomir for transfection, followed by a functional assessment using multiple experimental approaches. The EdU incorporation assay demonstrated that AGAPIR overexpression significantly promoted SCMECs proliferation, whereas functional inhibition of AGAPIR significantly reduced their proliferation (**Figure** **4**A,B). Migration was assessed through wound healing at various time points and transwell assays after transfection with AGAPIR agomir/antagomir. Consistent with the proliferation assay results, the overexpression of AGAPIR resulted in an increased migration of bEnd.3 cells (Figure 4C–F). Tube formation assays further confirmed the pro‐angiogenic effect, showing improved tubular network formation in AGAPIR‐overexpressing bEnd.3 cells (Figure 4G,H). Given the relatively low basal expression of AGAPIR in quiescent endothelial cells (Figure 3E,F), suppression of AGAPIR function by antagomir demonstrated only modest attenuation of migratory activity and tube‐forming capacity in bEnd.3 cells (Figure 4D,F,H). To evaluate endothelial barrier function using an OGD model, immunofluorescence staining revealed that OGD treatment markedly reduced Claudin‐5 and ZO‐1 fluorescence intensity, whereas OGD/R combined with AGAPIR agomir administration effectively restored the expression of these tight junction proteins (Figure 4I,J). Collective results from proliferation, migration, tube formation, and permeability assessments consistently indicated that AGAPIR enhanced the angiogenic potential of endothelial cells in vitro.

### 2.4 AGAPIR Modulates Ubiquitinated Protein Degradation and Targets USP18

**Figure 5:**
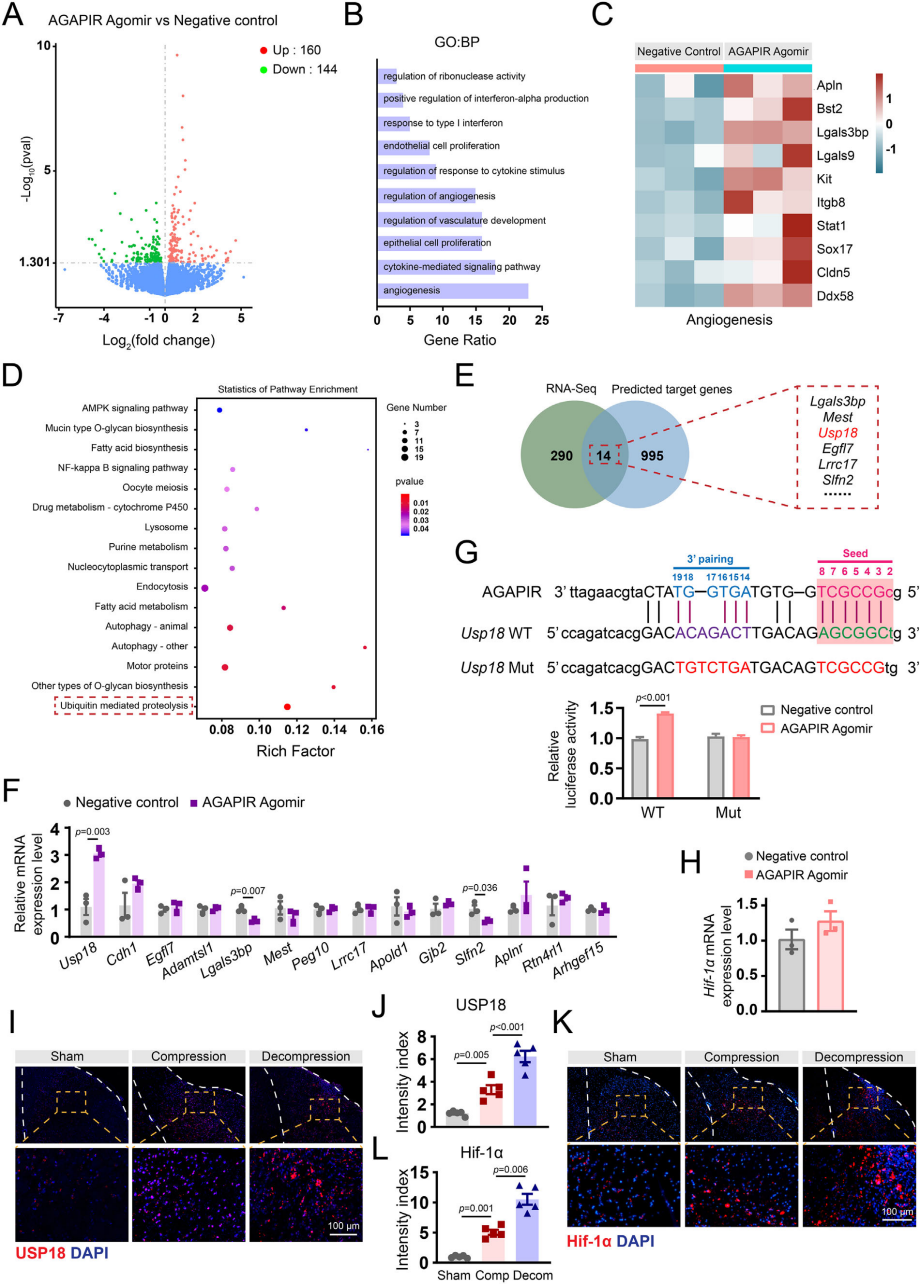
AGAPIR interacts with USP18 mRNA and upregulates its expression. A) Volcano plot of differentially expressed genes (DEGs) in SCMECs treated with AGAPIR agomir versus negative control. B) Gene Ontology (GO) analysis of AGAPIR agomir‐induced DEGs in SCMECs highlights significant enrichment of angiogenesis‐associated terms. C) Heatmap displaying expression profiles of SCMECs transduced with AGAPIR agomir or negative control. D) KEGG pathway analysis of predicted AGAPIR target genes. E) Venn diagram illustrating overlap between AGAPIR‐regulated genes (RNA‐seq) and bioinformatically predicted AGAPIR‐binding genes. F) Quantitative real‐time polymerase chain reaction validation of sequencing‐identified DEGs predicted to bind AGAPIR (n = 3/group). Data were analyzed using two‐tailed Student's *t*‐test. G) Bioinformatic prediction using miRanda software identified a putative AGAPIR‐binding site in wild‐type USP18 (USP18‐WT), with a corresponding mutant sequence (USP18‐Mut) designed for validation. The interaction between AGAPIR and USP18 was validated using a dual‐luciferase reporter assay with USP18‐WT and USP18‐Mut in AGAPIR agomir and control cells (n = 3/group). Data were analyzed using two‐tailed Student's *t*‐test. H) HIF‐1α mRNA levels in SCMECs with or without AGAPIR agomir treatment (n = 3/group). Data were analyzed using two‐tailed Student's *t*‐test. I–L) Immunofluorescence staining and quantitative analysis of USP18 and HIF‐1α in spinal cord tissues from sham, compression, and decompression groups. Mean fluorescence intensity was quantified in region of interest (n = 5/group). Statistical analysis: one‐way ANOVA with Bonferroni (J) or Dunnett's T3 (L) post hoc test.

To investigate the molecular mechanisms underlying AGAPIR‐mediated angiogenesis, we performed RNA sequencing on SCMECs transfected with the AGAPIR agomir or negative control. Following the removal of low‐intensity signals and normalization of the data, 304 differentially expressed mRNAs (160 upregulated and 144 downregulated) were identified using volcano plot analysis (**Figure** **5**A). Subsequent GO bioinformatics analysis revealed that AGAPIR agomir treatment significantly enhanced angiogenesis‐related processes (Figure 5B). Consistent with these findings, the heatmap analysis (Figure 5C) revealed significant elevation of angiogenesis‐related gene expression in AGAPIR agomir‐treated SCMECs relative to negative control, providing additional molecular evidence for AGAPIR's angiogenic‐promoting properties. Next, bioinformatics analysis was employed to predict the potential target genes of AGAPIR, followed by Kyoto Encyclopedia of Genes and Genomes (KEGG) pathway enrichment analysis of these predicted targets. Our analysis predicted 1009 AGAPIR target genes and identified 16 significantly enriched pathways, with ubiquitin‐mediated proteolysis being the most prominent (Figure 5D). These data collectively suggest that AGAPIR may mechanistically regulate angiogenesis through modulation of ubiquitin‐dependent protein degradation pathways. By combining RNA‐seq data with predicted AGAPIR targets, we identified 14 candidate genes. Analysis showed USP18—a deubiquitinating enzyme controlled by ubiquitination (Figure 5E). RT‐qPCR confirmed USP18 had the most significant expression change after AGAPIR agomir treatment compared to controls (Figure 5F), suggesting USP18 is a direct target of AGAPIR and may regulate the ubiquitin‐proteasome pathway.

Similar to miRNAs, the specific mechanisms by which piRNAs exert their effects (slicing or binding without slicing) are significantly dependent on the degree of base pairing between piRNAs and their intended mRNA targets.^[^ ^24^ ^]^ However, the specific extent of base pairing required for piRNAs remains unclear. Previous reports have indicated that piRNAs target mRNAs through seed sequence‐based interactions, specifically a perfect match in nucleotides 2–8, and additional base pairing in nucleotides 14–19.^[^ ^35^, ^36^ ^]^ Therefore, we investigated whether AGAPIR possesses a putative binding site within the USP18 mRNA using miRanda, a microRNA‐specific target detection algorithm. Our computational analysis predicted complementary base‐pairing interactions between AGAPIR and USP18, with subsequent experimental validation through dual‐luciferase reporter assays demonstrating that AGAPIR agomir significantly enhanced luciferase activity in USP18‐WT reporter vector (Figure 5G). This regulatory effect was abolished in USP18‐Mut vectors, confirming USP18 as a direct functional target of AGAPIR in SCMECs.

It is well established that HIF‐1α regulation through ubiquitination is closely linked to modulation of angiogenesis. To determine whether AGAPIR regulates HIF‐1α transcriptionally, we performed RT‐qPCR in SCMECs. Results showed that HIF‐1α mRNA levels remained unchanged upon AGAPIR overexpression (Figure 5H), excluding transcriptional regulation of HIF‐1α by AGAPIR. Interestingly, both USP18 and HIF‐1α were highly expressed in the spinal cord tissue of the compression group, with their expression levels further elevated in the decompression group compared to the compression group (Figure 5I–L). These findings suggest that AGAPIR may regulate HIF‐1α stabilization via the deubiquitinating activity of USP18 rather than influencing HIF‐1α gene expression.

### 2.5 AGAPIR Promotes Angiogenesis via Modulation of the USP18/HIF‐1α Axis

**Figure 6:**
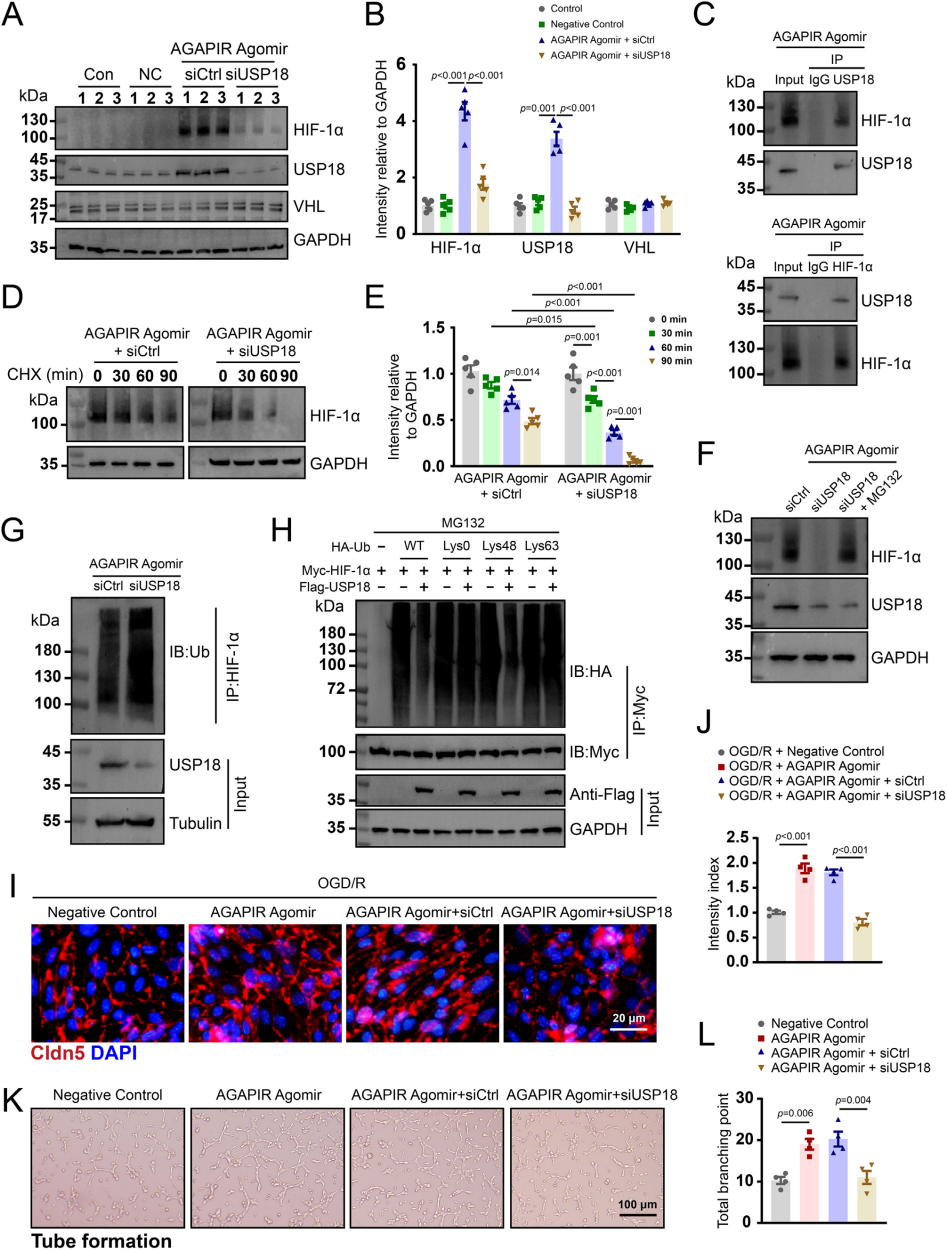
AGAPIR promotes angiogenesis by regulating the USP18/HIF‐1α axis in SCMECs. A) Representative western blot images of HIF‐1α, USP18, VHL, and GAPDH in SCMECs treated with: Control, Negative control, AGAPIR agomir + siCtrl, AGAPIR agomir + siUSP18. B) Quantitative analysis of protein levels normalized to GAPDH (n = 5/group). C) Endogenous protein associations were examined in SCMEC lysates by immunoprecipitation with anti‐USP18 or anti‐IgG and assessed by western blots with indicated antibodies to evaluate HIF‐1α. The interactions were verified through immunoprecipitation using anti‐HIF‐1α or anti‐IgG antibodies, followed by examination via western blot analysis with anti‐USP18 antibodies. D) HIF‐1α protein stability assay: western blot analysis of AGAPIR agomir + siCtrl versus AGAPIR agomir + siUSP18 SCMECs treated with cycloheximide (10 µg mL^−1^ for indicated durations. E) Quantification of HIF‐1α degradation kinetics (n = 5/group). F) Representative western blots images show the protein expression level of HIF‐1α, USP18 in SCMECs treated with siUSP18 or siUSP18 + MG132 (a proteasome inhibitor, 20 µM). G) Cell lysates from SCMECs transfected with siCtrl or siUSP18 following the treatment with MG132 and measured by western blots. H) SCMECs were transfected with Myc‐HIF‐1α, FLAG‐USP18, and the indicated HA‐Ub Lys0, Lys48‐only, or Lys63‐only plasmids, and then the HIF‐1α ubiquitylation linkage was analyzed. I) Representative immunofluorescence images and quantification J) of Claudin‐5 in SCMECs in each group (n = 4/group). K) Representative images of tube formation assay in bEnd.3 cells after negative control, AGAPIR agomir, AGAPIR agomir + siCtrl or AGAPIR agomir + siUSP18 treatment and L) quantification analysis of total branching points (n = 4/group). Statistical analysis: one‐way ANOVA with Bonferroni (B, E, J, L) or Dunnett's T3 (B) post hoc test.

To further verify the modulation of AGAPIR on USP18 and HIF‐1α, SCMECs were transfected with AGAPIR agomir alone or in combination with anti‐USP18 siRNA or control siRNA. As shown in **Figure** **6**A, western blot analysis revealed that AGAPIR agomir markedly upregulated USP18 and HIF‐1α expression, whereas USP18 siRNA effectively attenuated this AGAPIR‐mediated induction. Notably, AGAPIR agomir did not alter the expression of VHL, a critical component of the E3 ubiquitin ligase complex responsible for HIF‐1α degradation (Figure 6A,B). These observations suggest that AGAPIR may stabilize HIF‐1α through USP18‐mediated deubiquitination. Next, we used the co‐immunoprecipitation assays to investigate whether USP18 binds to HIF‐1α protein. As illustrated in Figure 6C, HIF‐1α was precipitated in SCMECs by USP18 following AGAPIR treatment, whereas no precipitation occurred with control immunoglobulin G (IgG). Additionally, reverse co‐immunoprecipitation confirmed that USP18 was significantly precipitated by HIF‐1α. Together, these different approaches clearly established that USP18 binds to HIF‐1α.

To elucidate USP18's role in HIF‐1α protein stability, we investigated the impact of USP18 knockdown on endogenous HIF‐1α stability in SCMECs using cycloheximide (CHX), a protein synthesis inhibitor. Following AGAPIR agomir treatment, CHX‐induced HIF‐1α degradation was significantly accelerated in USP18‐knockdown cells, demonstrating that USP18 is essential for maintaining HIF‐1α stability and regulating its degradation (Figure 6D,E). Consistently, blocking protein degradation with a proteasome inhibitor, MG132, resulted in HIF‐1α accumulation after knockdown of USP18 in SCMECs (Figure 6F). Together, these findings establish that USP18 maintains HIF‐1α stability through the ubiquitin‐proteasome pathway. We also observed abundant ubiquitination of HIF‐1α in the knockdown of USP18 SCMECs following AGAPIR treatment, but not in the siRNA control (Figure 6G). Lys48‐ and Lys63‐linked chains represent the two major forms of polyubiquitin chains. Lys48‐linked polyubiquitin chains primarily serve as targeting signals for proteasomal degradation, whereas Lys63‐linked polyubiquitin chains are involved in various cellular processes that do not depend on proteasome‐mediated degradation pathways. Accordingly, we investigated which type of polyubiquitin modification on the HIF‐1α protein is regulated by USP18. Our investigations demonstrated that USP18 specifically disassembled Lys48‐linked polyubiquitylation of HIF‐1α without affecting its Lys63‐linked counterpart. Collectively, these findings establish USP18 as a critical regulator of HIF‐1α stability through selective removal of Lys48‐linked ubiquitin chains, thereby preventing proteasomal degradation.

Given the finding that USP18 interacts with and stabilizes HIF‐1α protein, we next explored whether USP18 regulates angiogenesis in vitro. SCMECs were transfected with the AGAPIR agomir following USP18 siRNA treatment and subjected to OGD for 6 h followed by reoxygenation (OGD/R) for 12 h. As shown in Figure 6I,J, USP18 silencing abolished AGAPIR‐induced Claudin‐5 upregulation. Tube formation assays further confirmed that USP18 deficiency significantly impaired the pro‐angiogenic capacity of AGAPIR treated cells (Figure 6K,L). These collective results demonstrate that USP18 serves as an essential mediator of AGAPIR angiogenic effects by stabilizing HIF‐1α and maintaining endothelial functionality.

### 2.6 AGAPIR Improves Angiogenesis and Neurological Function Recovery in DCM Mice Following Surgical Decompression

**Figure 7:**
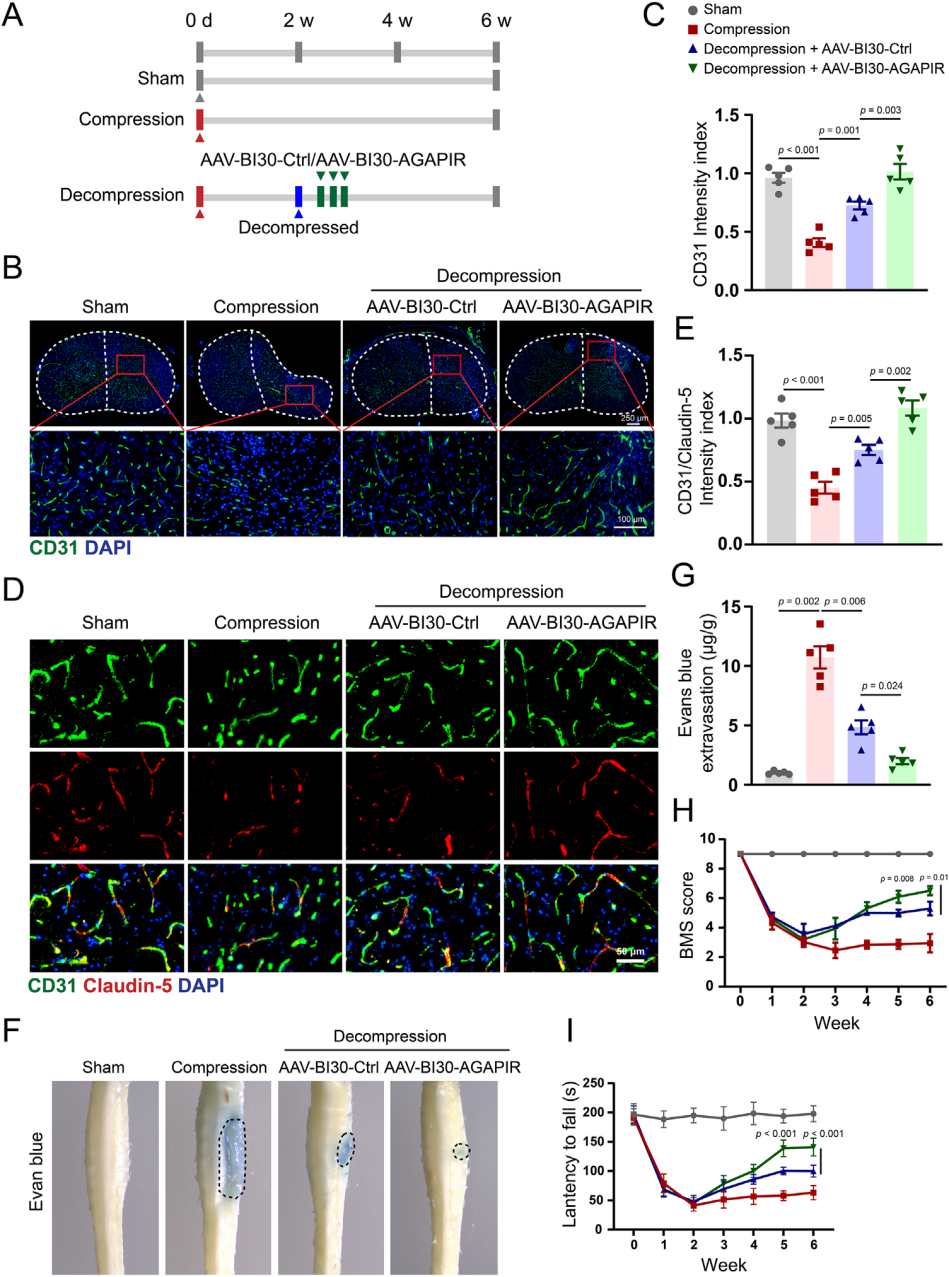
AGAPIR enhances post‐decompression angiogenesis and functional recovery in DCM. A) Experimental timeline of DCM mouse model establishment and interventions. B) Representative immunofluorescence staining of CD31 in spinal cord lesions and C) quantification of mean fluorescence intensity of CD31 in region of interest of spinal cord. D) Double‐immunofluorescence staining for CD31 (green) and Claudin‐5 (red) in frozen sections and E) quantification of the number of CD31^+^Claudin‐5^+^ cells in a specific area of spinal cord. F) Representative images and G) quantification of Evans blue leakage in the mouse spinal cord injury site. H) Distribution of the BMS scores in sham, compression, decompression + AAV‐BI30‐Ctrl, decompression + AAV‐BI30‐AGAPIR groups throughout the 6‐week period (n = 5/group). I) Motor coordination was quantified weekly by fall latency on an accelerating rotarod for all experimental groups. (n = 6/group). One‐way ANOVA with Bonferroni (C, E, H, I) or Dunnett's T3 (G, H) post hoc tests. Data represent mean ± SEM.

DCM mouse models were developed to investigate the effects of AGAPIR on angiogenesis in vivo. In the decompression group, mice received either a negative control or AAV‐BI30‐AGAPIR treatment for 3 days, beginning on the third day after decompression surgery (**Figure** **7**A). The AAV‐BI30 vector is distinguished by its ability to specifically and efficiently transduce endothelial cells across the central nervous system. The AAV‐BI30 construct the fluorescent reporter mCherry was intravenously administered via tail vein injection in mice. Immunofluorescence analysis revealed robust mCherry signal was restricted to spinal cord endothelial cells (78 ± 7% targeting efficiency, n = 3), while maintaining structural integrity of major organs, as evidenced by comprehensive histopathological assessment (Figure S4A–C, Supporting Information). This dataset establishes AAV‐BI30 as a precision vector for spinal cord endothelial cell‐specific delivery in vivo. Immunofluorescence staining revealed that the intensity of CD31‐positive endothelial cells within the decompressed spinal cord was significantly higher in the AAV‐BI30‐AGAPIR‐treated group than in the negative control group (Figure 7B,C). This finding suggests that AGAPIR overexpression promotes angiogenesis in the decompression mouse model.

To evaluate the impact of AGAPIR on blood‐spinal cord barrier (BSCB) integrity following decompression, we used immunofluorescence staining and evaluated Evans blue extravasation as an indicator of BSCB permeability. The results indicated that the fluorescence intensity of Claudin‐5 in the spinal cord tissue was notably higher in decompressed mice treated with AAV‐BI30‐AGAPIR than in those receiving negative control treatment (Figure 7D,E). Moreover, the area of Evans blue extravasation was significantly increased in mice with compression. In contrast, treatment with AAV‐BI30‐AGAPIR markedly reduced Evans blue leakage compared to the negative control group, indicating that AGAPIR has the capacity to restore BSCB integrity in decompressed mice (Figure 7F,G).

Next, we assessed limb motor function to evaluate the therapeutic effects of AGAPIR on neurological recovery after decompression. All injured animals exhibited a baseline BMS score of 4 at 1‐week post‐injury. Notably, starting from the third week post‐decompression, AAV‐BI30‐AGAPIR‐treated mice demonstrated significantly enhanced motor recovery compared to negative controls (Figure 7H). Critically, this improvement coincided with better balance and motor coordination in the same treatment group during rotarod testing (Figure 7I). Additionally, immunofluorescence analyses revealed AGAPIR‐mediated augmentation of neurofilament density in post‐decompression spinal cord tissues (Figure S5A,B, Supporting Information), correlating axonal preservation with functional improvement. These data demonstrate that AGAPIR administration substantially promotes neurological functional restoration in murine DCM models after surgical decompression.

## 3 Discussion

In this study, we used a novel, reproducible, and clinically relevant mouse model of chronic spinal cord compression at the C4–C6 level, which is the most common site of spinal cord compression in patients with DCM.^[^ ^37^ ^]^ This mouse model of chronic and progressive spinal cord compression is consistent with the clinical pathology of patients with DCM. Moreover, this mouse model provides the added advantage of using genetically engineered mice for future studies, an improvement over previous models that often relied on rats or larger animal models. Unlike acute traumatic spinal cord injury, chronic compression does not cause severe hemorrhage or acute disruption of the BSCB.^[^ ^1^ ^]^ Our data showed progressive ischemia during chronic compression of the spinal cord, which resulted in functional deficits in mice. Surgical decompression is the mainstay of treatment for DCM, as it can halt the progression of spinal cord compression and improve functional impairment. We found that spinal cord decompression was accompanied by increased angiogenesis and improved neurological function. Consistent with these observations, surgical decompression is often effective, with > 80% of patients with DCM reporting significant neurological improvement after surgery.^[^ ^2^, ^4^ ^]^ Nonetheless, some patients may experience neurological deterioration and complications, mainly due to severe neurological damage that exists prior to decompression. To date, the molecular mechanisms governing functional improvement after decompression are poorly understood, and there are no effective medical therapies that directly restore neurological function.^[^ ^5^ ^]^ Thus, novel therapeutic approaches are urgently required to treat the neurological deficits in DCM.

In this study, we identified an angiogenesis‐associated piRNA, AGAPIR, which plays a pro‐angiogenic role in the spinal cord following surgical decompression by modulating the USP18/HIF‐1α axis. Previous reports have suggested that impaired spinal cord perfusion is a major pathophysiological feature of DCM. Ample evidence has confirmed that promoting angiogenesis and restoring the blood supply essential for cell survival contribute to the restoration of locomotor function following spinal cord injury.^[^ ^38^, ^39^ ^]^ Our data demonstrate that AGAPIR promotes blood vessel formation, decrease the permeability of the BSCB, and further improves the recovery of neurological function in DCM mice after spinal cord decompression. To the best of our knowledge, this is the first study to provide evidence that increased angiogenesis can improve neurological function in mice with DCM following decompression.

piRNAs are increasingly recognized as key regulators of various developmental and pathophysiological processes, but their functional role in DCM remains largely uncharacterized. Recent studies have implicated dysregulated piRNA pathways in neurodevelopmental and neurodegenerative disorders, such as Alzheimer's disease,^[^ ^40^, ^41^ ^]^ Parkinson's disease,^[^ ^42^, ^43^ ^]^ and autism spectrum disorders.^[^ ^44^ ^]^ For instance, altered piRNA expression profiles have been observed in brain tissues of patients with Alzheimer's disease, potentially contributing to aberrant tau phosphorylation and amyloid‐β accumulation through interactions with synaptic plasticity‐related genes. Emerging evidence has highlighted their role in maintaining neuronal integrity by suppressing retrotransposon‐driven genomic instability, which may exacerbate neuroinflammation and oxidative stress.

Here, we demonstrate for the first time that DQ703900 (piR‐mmu‐119222) is highly expressed in the spinal cord of mice following decompression and promotes angiogenesis in vitro and in vivo. Overexpression of DQ703900 in SCMECs promoted proliferation, migration, and tube formation and significantly increased the protein expression levels of Claudin‐5 and ZO‐1. Therefore, DQ703900 was renamed AGAPIR. This exploration highlights a potential novel application of piRNAs in enhancing angiogenesis, which may be critical for improving neurological outcomes following surgical decompression in DCM patients.

Previous studies have shown that piRNAs function through a wide variety of molecular mechanisms. In animal germ cells, piRNAs guide the PIWI proteins to silence transposable elements and maintain genome integrity.^[^ ^45^ ^]^ piRNA‐30473 interacts with the 3′UTR of the *Wtap* gene and upregulates *Wtap* expression by regulating m6A RNA methylation in diffuse large B‐cell lymphoma.^[^ ^46^ ^]^ piRNA‐1742 regulates the stability of USP8 mRNA by binding directly to hnRNPU, which acts as a deubiquitinating enzyme that inhibits the ubiquitination of MUC12 and promotes the development of malignant renal cell carcinoma.^[^ ^47^ ^]^ Our results demonstrate that AGAPIR directly binds USP18 mRNA, upregulating its expression and consequently stabilizing HIF‐1α through USP18‐mediated deubiquitination. This non‐canonical regulatory mechanism—distinct from typical PIWI‐ related gene silencing pathways—supports our hypothesis that AGAPIR functions as a guide RNA that recruits PIWIL1‐containing complexes to regulate target gene expression.^[^ ^16^, ^46^ ^]^ Critically, knockdown of USP18 in SCMECs blocked the pro‐angiogenic activity of AGAPIR and increased HIF‐1α ubiquitination and degradation. These findings suggest that deubiquitination mediates the regulation of angiogenesis related to spinal AGAPIR. However, the specific mechanism through which AGAPIR increases USP18 levels requires further investigation.

There is growing evidence that the deubiquitinase USP18 plays a key role in the regulation of microglial activation, suppression of apoptosis, reactive astrocytes, and endothelial cell regulation by abolishing the ubiquitination of associated proteins.^[^ ^48^, ^49^, ^50^ ^]^ Our current data revealed that AGAPIR specifically bound to *Usp18* and increased its protein expression. Moreover, protein expression of USP18 increased during the process from spinal cord compression to decompression. Interestingly, the expression of HIF‐1α in spinal cord samples undergoing compression and decompression showed a similar trend. Furthermore, our data demonstrated that overexpression of AGAPIR stabilized HIF‐1α protein by increasing USP18 levels. Specifically, USP18 directly deubiquitinates and stabilizes HIF‐1α to protect it against ubiquitination and proteosome‐mediated degradation in SCMECs. Increased HIF‐1α activity is a key regulator of angiogenesis in active endothelial cells.^[^ ^29^ ^]^ As a result, our current experiment may provide new insights to the understanding of the interaction between USP18 and HIF‐1α.

Surgical decompression alleviates mechanical compression‐induced ischemia, allowing restoration of blood flow to the spinal cord. Increased vascularization after decompression enhances oxygen and nutrient delivery to ischemic neurons and oligodendrocytes, thereby mitigating hypoxic damage and supporting cellular repair.^[^ ^33^, ^51^ ^]^ Our study demonstrated that AGAPIR promoted angiogenesis, which was correlated with improved neurofilament density (Figure S5, Supporting Information). This aligns with clinical evidence that elevated spinal cord blood flow post‐decompression predicts better neurological recovery in patients with DCM.^[^ ^33^, ^52^ ^]^ Chronic compression disrupts the BSCB and exacerbates inflammation. Post‐decompression angiogenesis restores the endothelial integrity and BSCB function, thereby reducing neurotoxic molecular infiltration and inflammation. Studies have indicated that angiogenic mediators such as VEGF‐A are critical for endothelial repair.^[^ ^29^ ^]^ While recombinant VEGF administration carries inherent vascular leakage risks due to its potent permeability‐enhancing properties.^[^ ^53^, ^54^, ^55^ ^]^ In contrast, AAV‐BI30‐mediated AGAPIR overexpression strategy provides a targeted alternative that not only enhances BSCB repair to support neuronal survival but also offers key translational advantages: AGAPIR's piRNA nature enables rapid in vitro synthesis with straightforward modifications for precise spinal cord endothelial targeting; and CNS‐specific AAV‐BI30 delivery ensures spatially restricted expression, avoiding systemic complications. Collectively, these attributes—combined with its mechanistic role in vascular repair—position AGAPIR as a promising clinical candidate for spinal cord injury recovery.

Newly formed vessels secrete neurotrophic factors (e.g., BDNF and GDNF) and provide structural scaffolds for axonal regrowth. Revascularization also facilitates the removal of metabolic waste and inflammatory cytokines, thereby creating a microenvironment conducive to neural plasticity.^[^ ^56^ ^]^ In our study, recovery of neurofilament density paralleled angiogenesis (Figure S5, Supporting Information), suggesting that vascular regeneration directly supports axonal repair. Although delayed decompression exacerbates ischemia‐reperfusion injury by promoting oxidative stress and apoptosis, timely intervention facilitates controlled revascularization and enhances functional recovery through pro‐angiogenic effects in preclinical models.^[^ ^57^, ^58^ ^]^

Our study had some limitations. First, the lack of clinical data from laboratory samples was a potential limitation. Recent reports have shown that circulating miRNAs may potentially serve as targets for future therapeutic interventions or diagnostic/prognostic testing in patients with DCM.^[^ ^7^ ^]^ Future studies are recommended to investigate piRNA levels in the blood or cerebrospinal fluid. Second, although prolonged compression duration did not worsen short‐term symptoms in our DCM mouse model, it correlated with long‐term functional deficits after surgical decompression, with early decompression demonstrating superior neural recovery compared to delayed decompression.^[^ ^57^ ^]^ Our model offers advantages in stability, reproducibility, and moderate modeling duration, but future refinements—such as incorporating engineered materials to decelerate volumetric expansion and extend time‐to‐peak compression (e.g., from 2 to 4–6 weeks)—would better mimic chronic DCM progression. These improvements could enhance the model's pathological relevance and provide a platform for evaluating therapeutic strategies like AGAPIR in delayed decompression scenarios.

## 4 Conclusion

**Figure 8:**
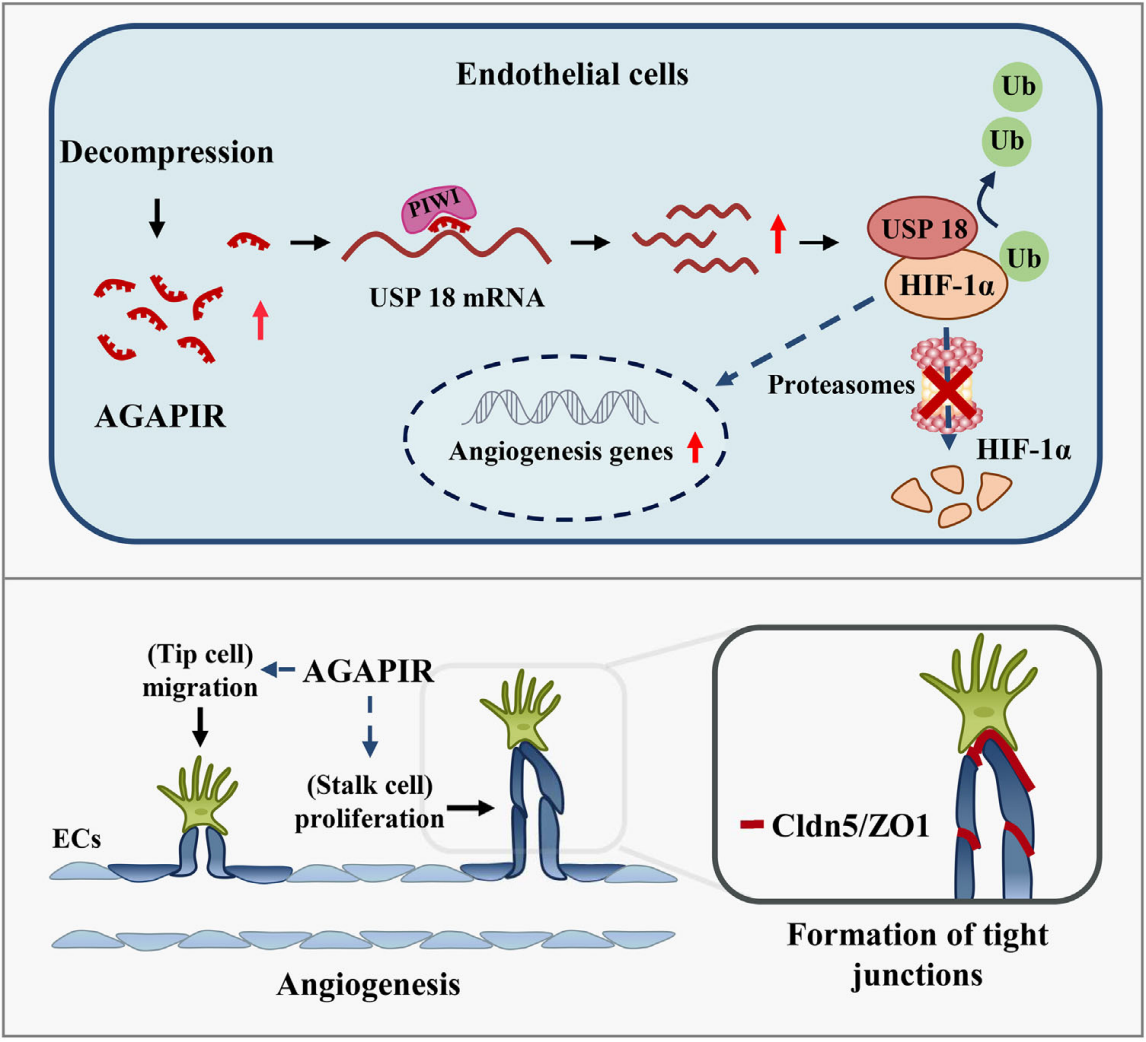
The molecular mechanism by which AGAPIR potentiates angiogenesis via the USP18/HIF‐1α axis. Under decompression conditions, AGAPIR expression is upregulated and directly interacts with USP18 mRNA to enhance its transcriptional output. USP18, functioning as a deubiquitinase, stabilizes HIF‐1α by inhibiting its proteasomal degradation, leading to HIF‐1α accumulation. Elevated HIF‐1α activates hypoxia‐responsive pathways, including transcriptional upregulation of pro‐angiogenic factors, which drive endothelial cell migratory dynamics and proliferative expansion, culminating in neovascularization.

In conclusion, the results presented in this study demonstrated a pro‐angiogenic role of AGAPIR, USP18, and HIF‐1α in regulating SCMECs. Furthermore, through gain‐ and loss‐of‐function studies, we demonstrated that the beneficial effects of AGAPIR overexpression in DCM mice were associated with increased angiogenesis in the spinal cord after surgical decompression (**Figure** **8**). These results may serve as a roadmap for future clinical studies to evaluate the potential therapeutic effects of AGAPIR overexpression in patients with DCM following surgical decompression.

## 5 Experimental Section

### Chronic Compressive Spinal Cord Injury Model and Treatments

All animal care and experimental procedures adhered to the guidelines approved by the Institutional Animal Care and Use Committee (IACUC) of Shenzhen People's Hospital (approval number: AUP‐230809‐LHQ‐0379‐01). Male C57BL/6 mice (10 weeks old, body weight 25–30 g) and male Sprague‐Dawley rats (4 months old, body weight 320–350 g) were obtained from the Guangdong Medical Laboratory Animal Center. All animals were housed in specific pathogen‐free ventilated cages maintained at 23 ± 2 °C with 55% humidity and a 12‐h light/dark cycle, with free access to food and clean water.

The chronic compressive spinal cord injury mouse model of DCM was adapted from a rat DCM model previously established in the laboratory.^[^ ^59^ ^]^ Briefly, male C57BL/6 mice were anesthetized using pentobarbital sodium (45 mg kg^−1^) via intraperitoneal injection. The C7 spinous process was used as a reference point after exposure. Forceps were used to gently separate the muscles attached to the C6 lamina, thereby minimizing the risk of bleeding before removing the C6 lamina. A polymer sheet, specifically (1⟶4)‐3,6‐anhydro‐a‐l‐galactopyranosyl (1⟶3)‐β‐D‐galactopyranan was implanted into the space underneath C4‐C6 laminae,^[^ ^60^ ^]^ which is the most common site for spinal cord compression in patients with DCM.^[^ ^61^ ^]^ Spinal cord compression is achieved through polymer swelling due to fluid absorption. The sheet can absorb tissue fluid and gradually expand into an ellipsoidal cylinder.^[^ ^62^ ^]^ In the sham group, the C6 lamina was excised without implantation, whereas in the decompression group, the polymer was removed 2 weeks postoperatively. Following the operation, the incision was closed, the skin was disinfected, and the mice were placed in an incubator for recovery. For the chronic compressive spinal cord injury rat model, a polymer sheet was implanted in the space underneath the C4–C6 laminae.

To investigate the pro‐angiogenic effect of AGAPIR in mice, mice that had undergone decompression were randomly assigned to receive either 200 µl of AAV‐BI30‐AGAPIR virus (≈1 × 10^11^ particles, ShanDong ViGene Co., Ltd., China) or AAV‐BI30‐Ctrl virus (≈1 × 10^11^ particles) via the tail vein starting on day 3 post‐decompression. The AAV‐BI30 capsid is known for specific and efficient transduction of endothelial cells throughout the central nervous system.^[^ ^63^ ^]^ The mice were euthanized and the spinal cord tissue was collected for subsequent analysis.

### BMS

Following compression/decompression surgery, motor function was assessed weekly using the BMS system as previously described by two trained investigators who were blinded to the experimental design.^[^ ^64^ ^]^ During the assessment, the mice were placed in an open area for four min to evaluate all visually discernible aspects of motor recovery. The BMS score ranges from 0 to 9, indicating complete paralysis (0) to normal movement (9), and is based on various factors, including limb movement, limb joint movement, bilateral limb coordination, plantar stepping, weight support, trunk stability, and tail position.

### Rotarod Test

Motor coordination was assessed weekly using a rotarod apparatus (Model KW‐6C, NJKEWBIO, Nanjing, China). The apparatus featured a 30 mm diameter rod with a rippled surface to minimize slippage. Each mouse underwent one practice trial followed by two formal trials with 15 min interval between them. The testing protocol began with a 20 s acceleration to 4 rpm, followed by a linear acceleration from 4 to 40 rpm over 280 s.^[^ ^65^, ^66^ ^]^ Fall latency was automatically recorded, and the mean value was calculated from the two formal trials.

### Histological Analyses

For immunofluorescence staining, tissues were fixed in 4% (w/v) paraformaldehyde overnight, followed by incubation in 20% sucrose and 2% polyvinylpyrrolidone (PVP) at 4 °C for 24 h, and subsequently embedded in OCT. Fifteen‐micrometer‐thick sections were immersed in PBS, permeabilized using 0.3% Triton X‐100 in PBS (0.3% PBST) for 20 min, and then incubated in a blocking solution consisting of 10% normal goat serum and 0.3% PBST for 30 min. The sections were incubated overnight at 4 °C with the following primary antibodies: CD31/PECAM‐1 Alexa Fluor 488‐conjugated antibody (#FAB3628G‐100, R&D Systems, 1:100), Ki67 (#ab16667, Abcam, Cambridge, UK, 1:200), α‐smooth muscle actin (D4K9N) (#19 245, Cell Signaling Technology, Massachusetts, USA, 1:200), von Willebrand factor (#GB11020, Servicebio, 1:200), Claudin‐5 (E8F3D) (#49 564, Cell Signaling Technology, 1:100), ZO1 (#ab96587, Abcam, 1:100), USP18 (E9K4X) (#53 229, Cell Signaling Technology, 1:100), and HIF‐1α (D1S7W) (#49 564, Cell Signaling Technology, 1:100). After washing three times with PBS, sections were incubated for 1 h at room temperature in darkness with Dylight 594‐conjugated goat anti‐rabbit IgG (#A23420, Abbkine, 1:200) and Dylight 488 goat anti‐rabbit IgG (#A23220, Abbkine, 1:200). Nuclei were counterstained with 4′,6‐diamidino‐2‐phenylindole (DAPI, #ab104139, Abcam).

For immunofluorescence staining in cell cultures, cells were grown on coverslips in 24‐well plates. Upon harvesting, cells were washed three times with PBS and fixed in 4% paraformaldehyde at room temperature for 15 min. Subsequently, cells were permeabilized in 0.3% PBST for 20 min. After an additional wash with PBS, cells were incubated in blocking solution for 30 min, followed by overnight incubation at 4 °C with Claudin‐5 (E8F3D) (#49 564, Cell Signaling Technology, 1:100) and ZO1 (#ab96587, Abcam, 1:100). The next day, cells were rinsed three times with 0.1% PBST and incubated with corresponding secondary antibody for 1 h. Nuclei were counterstained with DAPI. Images were acquired using a fluorescence microscope (IX73PIF, Olympus, Japan).

For hematoxylin and eosin (H&E) staining, tissues were fixed in 4% paraformaldehyde overnight, dehydrated through a graded ethanol series, and embedded in paraffin. Four‐micrometer‐thick sections were stained with H&E using a standard protocol. Images were captured using an Olympus CKX53 microscope.

### scRNA‐Seq and Data Processing

scRNA‐seq was conducted using spinal cord tissue from control and chronic spinal cord compression rats (n = 3 per group). The tissues were placed in a Petri dish on wet ice, pre‐treated with 1× PBS (devoid of RNase and Ca, Mg ions), and washed to eliminate blood stains, grease, and other contaminants from the tissue surface. Subsequently, the tissue was cut into pieces ≈0.5 mm^2^ in size and washed again with 1× PBS. These cleaned pieces were treated with a dissociation solvent (0.35% collagenase IV, 2 mg mL^−1^ papain, 120 Units mL^−1^ DNase I) and incubated for 20 min at 37 °C in a water bath shaker set at 100 rpm. To halt the dissociation process, PBS containing 10% fetal bovine serum was added. The resulting cell suspension was filtered through a 70–30 µm cell sieve and centrifuged at 300 g for 5 min at 4 °C. Following centrifugation, the cell pellet was collected and resuspended in 100 µl of 1× PBS (0.04% BSA). To remove erythrocytes, 1 mL of 1× erythrocyte lysis solution (MACS 130‐094‐183, 10×) was added and allowed to react at room temperature or on wet ice for 2–10 min. Following tissue dissociation, erythrocyte lysis, and dead cell removal, viable cells were obtained and resuspended in 100 µl of 1× PBS (with 0.04% BSA) to prepare the final cell suspension. Cell viability was determined using trypan blue staining, requiring a viability rate exceeding 85%. The number of cells was counted using either a hemocytometer or a Countess II Automated Cell Counter, achieving a concentration range of 700–1200 cells µL^−1^.

The prepared single‐cell suspension was loaded onto the 10× Chromium chip according to the guidelines provided for the 10× Genomics Chromium Single‐Cell 3′ kit (V3), aiming to capture ≈8000 cells. Complementary DNA (cDNA) amplification and library construction were carried out following standard protocols. LC‐Bio Technology (Hangzhou, China) performed the sequencing on an Illumina NovaSeq 6000 system, utilizing paired‐end sequencing with a read length of 150 bp, ensuring a minimum sequencing depth of 20 000 reads per cell.

The raw sequencing results were processed offline and converted to FASTQ format using bcl2fastq software (version 5.0.1). The scRNA‐seq data were aligned against a reference genome using CellRanger software, which facilitated the identification and quantification of cellular transcripts and individual cellular 3′ end transcripts within the sequenced samples. The expression profile matrix generated by CellRanger was subsequently imported into Seurat (version 4.1.0) for filtering low‐quality cells; this included setting thresholds such as a minimum of 500 expressed genes per cell and less than 25% mitochondrial gene expression. Hypergeometric testing was employed to conduct GO and KEGG enrichment analyses on differentially expressed genes identified across clusters. For cell type annotation, resources such as the SingleR database, the scCATCH database, and their own LC‐Marker were utilized. Additionally, some cells were re‐clustered based on SingleR's output to identify marker genes.

### RNA Isolation and Quantitative Real‐Time Polymerase Chain Reaction

Total RNA was extracted from the spinal cord tissues or cultured cells using AG RNAex Pro Reagent (#AG21102, Accurate Biotechnology, China) following a standard protocol. RNA concentration and purity were quantified using a Nanodrop spectrophotometer (NanoDrop2000c, Thermo Fisher Scientific, USA). For mRNA analysis, 1 µg of total RNA was reverse transcribed into cDNA using 5× Evo M‐MLV RT Master Mix (#A2A1152, Accurate Biotechnology). For piRNA analysis, 1 µg of total RNA was reverse transcribed using the miRNA First Strand cDNA Synthesis (Tailing Reaction) kit (#B532451, Sangon Biotech, China) according to the manufacturer's instructions. Real‐time PCR was conducted using 2× SYBR Green Pro Taq HS Premix (#A3A0689, Accurate Biotechnology) on a StepOnePlus quantitative PCR system (Applied Biosystems, Thermo Fisher Scientific) according to the manufacturer's guidelines. The relative expression levels of target genes were normalized to the housekeeping genes (*Gapdh* for mRNAs and *U6* for piRNAs) using the 2^−ΔΔCt^ method. The primers used in this study are detailed in the Table S1, Supporting Information.

### Isolation and Culture of SCMECs

SCMECs were isolated from 8–10‐week‐old male C57BL/6 mice following established protocols.^[^ ^34^ ^]^ Briefly, mice were sacrificed via cervical dislocation. Subsequently, the spinal cords were isolated, the meninges were detached, and the tissue was minced with a pipette until the medium appeared milky. DMEM (#11995‐065, Gibco, USA) containing 10 mg mL^−1^ collagenase type II (#C2‐28‐100MG, Sigma‐Aldrich, USA) was added and the mixture was digested for 1 h at 37 °C on an incubator shaker set to 180 rpm. The pellet was then collected and resuspended with a 1 mL pipette in 25 mL of bovine serum albumin‐DMEM (20%, w/v) ≈25 times, followed by centrifugation at 1000 ×g for 20 min at 4 °C to remove myelin. The resulting pellet was subsequently digested in DMEM containing 10 mg mL^−1^ collagenase/dispase (#10 269 638 001, Sigma‐Aldrich) for 1 h at 37 °C on an orbital shaker at 180 rpm. Finally, the pellets were purified using a Percoll gradient (#P8370, Solarbio, Beijing, China) and seeded onto cell culture plates in an endothelial cell medium containing 10 mg mL^−1^ puromycin (#HY‐K1057, MedChemExpress, USA). Treatment with puromycin facilitated the selection of murine SCMECs for subsequent experiments.

### RNA Sequencing and Data Analysis

Total RNA was extracted from spinal cord tissues 4 weeks post‐sham, compression, and decompression surgery using AG RNAex Pro Reagent (Accurate Biotechnology) for RNA sequencing. SCMECs were treated with either a negative control agomir or AGAPIR agomir for 24 h, followed by RNA extraction using the AG RNAex Pro Reagent. RNA was subsequently purified using RNAprep Pure (#DP441, TIANGEN, China), quantified using a NanoDrop ND‐1000, and assessed for quality using an Agilent 4200 TapeStation system to ensure RNA integrity. Sequencing libraries were generated using the Hieff NGS Ultima Dual‐mode RNA Library Prep kit (#12310ES, YEASEN, China), in accordance with the manufacturer's instructions, and sequenced on the Illumina Novaseq 6000 using the paired 150 platform. Differentially expressed genes (DEGs) were identified by having with a Log2 foldchange > 1 and an adjusted *p*‐value < 0.05.

### Western Blot Analysis

Tissues or cells were lysed using a radioimmunoprecipitation assay (RIPA) buffer (#G2002, Servicebio, China), which contained a phosphatase inhibitor cocktail (#G2007, Servicebio), proteinase inhibitors (#G2006, Servicebio), and phenylmethanesulfonyl fluoride (PMSF) (#G2008, Servicebio). The protein concentration was subsequently measured using the BCA Protein Assay Kit (#PC0020, Solarbio, China). Total protein was separated by sodium dodecyl‐sulfate polyacrylamide gel electrophoresis and transferred to a polyvinylidene fluoride (PVDF) Immobilon‐P membrane (#IPVH00010; Millipore, USA). Membranes were blocked with 5% skim milk powder in Tris‐buffered saline (TBS) containing 0.1% Tween‐20 (TBS‐T) prior to overnight incubation with primary antibodies at 4 °C. The primary antibodies utilized included: CD31 (PECAM‐1) (D8V9E) rabbit mAb (#77 699, Cell Signaling Technology, 1:1000), von Willebrand Factor (vWF) (#GB11020, Servicebio, 1:1000), USP18 (E9K4X) (#53 229, Cell Signaling Technology, 1:1000), HIF‐1α (D1S7W) (#49 564, Cell Signaling Technology, 1:1000), VHL rabbit pAb (#A0377, ABclonal, Wuhan, China, 1:1000), GAPDH rabbit polyclonal antibody (#ABL1012, Abbkine, Wuhan, China, 1:1000), and α‐tubulin monoclonal antibody (#ABL1080, Abbkine, 1:1000). The following day, the membranes were incubated with horseradish peroxidase (HRP)‐conjugated anti‐rabbit (#SSA004, Sino Biological, Beijing, China, 1:3000) or anti‐mouse (#SSA007, SinoBiological, 1:3000) secondary antibodies at room temperature for 1 h. All membranes were developed using Western Bright ECL HRP substrate (#G2161, Servicebio), and signals were detected using a chemiluminescence apparatus (ChemiScope 6100 Touch, Clinx, China).

### RNA Pull‐Down Assay

RNA pull‐down assays were performed using a PureBinding RNA‐Protein pull‐down Kit (#P0202, Geneseed Biotech, China) in accordance with the manufacturer's instructions. Briefly, cells were lysed in a capture buffer containing RNase and protease inhibitors for 10 min on ice. Following centrifugation, cell lysates were incubated with 100 µL of streptavidin magnetic beads and specific AGAPIR or negative control RNA probes for 1 h at 4 °C with rotation at 10 rpm. Biotin‐labeled AGAPIR (100 pmol) and negative control (NC) probes (100 pmol) were synthesized by GenePharma Co., Ltd. (China). The sequence of the AGAPIR probe was 5′‐GCGCCGCUGGUGUAUGUGGUAUCAUGCAAGAUU‐3′, which included biotin modifications. The RNA‐binding complexes were subsequently washed, eluted, and boiled at 100 °C for 10 min. The samples were subjected to electrophoresis for western blot analysis. The primary and corresponding secondary antibodies used were rabbit anti‐PIWIL1 (#bs‐0665R, Bioss, Beijing, China, 1:500), rabbit anti‐PIWIL2 (#bs‐3817R, Bioss, 1:500), rabbit anti‐PIWIL4 (#bs‐6853R, Bioss, 1:500), and HRP‐conjugated anti‐rabbit antibody (#SSA004, Sino Biological, 1:3000).

### FISH Assay

For the cell FISH assay, SCMECs were seeded onto crawling slides within 24‐well plates. After washing with PBS, 4% paraformaldehyde was added to each well, and the cells were fixed for 15 min at room temperature. The Cy3‐labeled AGAPIR and NC probes were synthesized by GenePharma. The sequence of the AGAPIR probe was: 5′‐AATCTTGCATGATACCACTACACCAGCGGCGC‐3′. The assay was subsequently performed using an RNA FISH kit (GenePharma, China) in accordance with the manufacturer's instructions. Briefly, a 4 µM probe mixture was prepared and denatured at 73 °C for 5 min. Following this, SCMECs were incubated in the probe mixture at 37 °C overnight for hybridization. Nuclei were counterstained with DAPI for 10 min.

For the frozen sections of spinal cord tissue, the slices were washed three times with PBS and subsequently incubated in a proteinase K solution at 37 °C for 20 min. Following gradient alcohol dehydration, slices were subjected to heat denaturation for 8 min. An 8 µM probe mixture was prepared and denatured at 73 °C for 5 min. The sections were hybridized at 37 °C overnight and, prior to counterstaining the nuclei with DAPI, incubated with the following antibodies at room temperature under light‐protected conditions: CD31/PECAM‐1 Alexa Fluor 488‐conjugated antibody (R&D Systems, 1:100), NeuN (D4G4O) XP rabbit mAb (Alexa Fluor 488 Conjugate) (#54 761, Cell Signaling Technology, 1:100), Iba1/AIF‐1 (E4O4W) XP rabbit mAb (Alexa Fluor 488 Conjugate) (#20 825, Cell Signaling Technology, 1:100), GFAP (GA5) mouse mAb (Alexa Fluor 488 Conjugate)(#3655, Cell Signaling Technology, 1:100), and Alexa Fluor 488 anti‐Olig2 antibody (#ab225099, Abcam,1:50). Images were captured using a fluorescence microscope (IX73PIF; Olympus, Tokyo, Japan). AGAPIR expression was analyzed using the ImageJ software (version 1.8.0, National Institutes of Health, USA).

### EdU (5‐ethynyl‐20‐deoxyuridine) Assay

The proliferation of SCMECs was assessed using the YF594 Click‐iT EdU Imaging Kits (#40276ES60, Yeasen, China). Cells were seeded at a density of 5 × 10^4^ per well in a 24‐well plate and subjected to various treatments. Following fixation and permeabilization, the cells were incubated with a 20 µM EdU working solution for 4 h. Subsequently, the cells were stained with the Click‐iT EdU reaction solution, and the cell nuclei were counterstained with DAPI for 5 min. Images were captured using an IX73PIF microscope (Olympus) and analyzed using the ImageJ software. The AGAPIR agomir and antagomir were synthesized by GenePharma, with the following sequences: agomir sense, 5′‐GCGCCGCUGGUGUAGUGGUAUCAUGCAAGAUU‐3′; agomir antisense, 5′‐UCUUGCAUGAUACCACUACACCAGCGGCGCUU‐3′; and antagomir, 5′‐AAUCUUGCAUGAUACCACUACACCAGCGGCGC‐3′.

### Scratch Wound Migration Assay

The bEnd.3 cells were purchased from Beijing Solarbio Science & Technology Co., Ltd., and cultured in DMEM supplemented with 10% fetal bovine serum (FBS) and 1% penicillin/streptomycin in a humidified incubator at 37 °C with 5% CO_2_. These cells were then transfected with either an agomir or an antagomir. Upon reaching ≈95% confluency, a cross‐shaped scratch was created in the monolayer of bEnd.3 cells using a 200 µl pipette tip. Following this, any suspended cells were removed by washing with PBS, and the cultures were maintained in DMEM without FBS supplementation to assess cell migration. The wound width was measured at 0 h, 6 h, and 12 h post‐scratching using a microscope (CKX53) and quantified using ImageJ software.

### Transwell Migration Assay

The bEnd.3 cells were seeded into a 6‐well plate under various stimulation conditions. Subsequently, the cells were digested and cultured in the upper transwell chamber (#3422, Corning, USA) using serum‐free medium, while 500 µL of DMEM supplemented with 10% FBS was added to the lower chambers. After 24 h incubation period, the bEnd.3 cells that had adhered to the upper surface of the polycarbonate films were carefully removed, and the cells that had migrated to the lower surface of the membranes were fixed with 4% paraformaldehyde for 10 min. These migrated cells were then stained with 0.1% crystal violet for 30 min. Cell images were captured using a microscope (CKX53).

### Tube Formation Assay

The bEnd.3 cells were seeded into a 6‐well plate with different stimulations. The cells were then digested and transferred onto a 48‐well plate that had been precoated with Matrigel (#356 230, Corning, USA). After being cultured for 4 h in the appropriate media, tubular structures formed by the cells were observed and captured using a microscope (CKX53). The ImageJ software was utilized to assess the number of branch points in five randomly selected microscopic fields.

### OGD and Reoxygenation

To mimic the hypoxic conditions and subsequent reoxygenation experienced by SCMECs during in vitro simulation of spinal cord compression and decompression, the OGD experiment was conducted as previously described.^[^ ^38^ ^]^ In brief, SCMECs were seeded into a 6‐well plate under various stimulation conditions. The cells were initially incubated in DMEM supplemented with 10% FBS in a cell incubator maintained at 5% CO_2_, 21% O_2_, and 37 °C with saturated humidity for 12 h, simulating the conditions for the sham operation group. For the compression group, the cells were incubated in DMEM without glucose or FBS and exposed to a hypoxic gas mixture consisting of 5% CO_2_, 0.1% O_2_, and 94.9% N_2_ for 6 h. Subsequently, for the decompression group, the cells were initially subjected to hypoxia for 6 h, followed by a return to normal culture conditions for an additional 12 h.

### Small Interfering RNAs

SCMECs were isolated and cultured in a 6‐well plate, settled overnight, and subsequently transfected with *Usp18* siRNA using Lipofectamine 2000 (#11 668 027, ThermoFish Scientific, USA) according to the manufacturer's instructions. Both the control and mouse‐specific *Usp18* siRNA were purchased from GenePharma. The following oligonucleotides of siRNA duplexes were used: *siUsp*18#1 5′‐ UAAGGUAGAGUUGAGCAGCTT‐3′; *siUsp*18#2, 5′‐ AUCAGGGACUCCUGCGUCCTT‐3′; *siUsp*18#3, 5′‐ UUUCCAAGGCGUCUUCUCCTT‐3′; *siUsp*18#4, 5′‐ AUUUCAGACUGUUCCUUGGTT‐3′.

### Immunoprecipitation

SCMECs were isolated and seeded in a 10‐cm plate under various stimulation conditions. The cells were subsequently lysed using NP‐40 lysis buffer (#P0013F; Beyotime, China) and the concentration of the protein lysate was quantified using a BCA Protein Assay Kit (Solarbio). ≈100 µg of the protein lysate was utilized as the input sample for western blot analysis. The remaining lysate was then incubated with protein A/G PLUS‐agarose beads (sc‐2003, SantaCruz Biotechnology, USA), pre‐coupled with a specific antibody, and rotated at 4 °C at 20 rpm overnight. The immunocomplexes were washed and boiled in loading buffer for western blot analysis.

### Luciferase Reporter Assay

To validate the direct targeting relationship between AGAPIR and USP18, complementary DNA sequences corresponding to the wild‐type (WT) or mutant (Mut) of USP18 containing putative AGAPIR binding sites were synthesized. These fragments were directionally cloned into the multiple cloning site downstream of the Renilla luciferase gene in the psiCHECK‐2 dual‐luciferase reporter vector (Hanbio Biotechnology, Shanghai, China), which constitutively expresses Firefly luciferase for normalization. The luciferase reporter plasmids were co‐transfected into SCMECs with a AGAPIR agomir or scrambled negative control (NC) using Lipofectamine 2000 (ThermoFish Scientific). Following 48 h incubation at 37 °C/5% CO_2_, cells were lysed with passive lysis buffer (Promega) and centrifuged at 12 000×g for 5 min at 4 °C. Luciferase activity was quantified using the Dual‐Luciferase Reporter Assay Kit (Hanbio Biotechnology) according to the manufacturer's protocol. Relative luciferase activity was calculated as the ratio of Renilla/Firefly luminescence and normalized to the NC group.

### In Vitro Ubiquitylation Assays

For preparation of ubiquitinated HIF‐1α as the substrate for the in vitro deubiquitination assay, SCMECs were transfected with Myc‐tagged HIF‐1α, FLAG‐tagged USP18, and hemagglutinin (HA)‐tagged ubiquitin and were treated with 20 µM MG132 for 8 h. Plasmids coding for HA‐tagged ubiquitin‐Lys63 and HA‐tagged ubiquitin‐Lys48 were purchased from Addgene (Watertown, Massachusetts, USA). Lysed proteins from cultured cells were precipitated and analyzed by immunoblotting using the following antibodies: anti‐HA‐tag (#3724, Cell Signaling Technology, 1:1000), anti‐Myc‐tag (#2276, Cell Signaling Technology, 1:1000), and anti‐FLAG‐tag (#F3165, Sigma‐Aldrich, 1:1000).

### Evans Blue Dye Assay

To assess the permeability of the blood‐spinal cord barrier (BSCB) in the mice, 0.2 mL of 2% Evans blue solution (#E2129, Sigma‐Aldrich) was administered intravenously via the tail vein. Following a 2 h period post‐injection, the mice were anesthetized using pentobarbital sodium (45 mg kg^−1^), and the spinal cord tissues at the injury site were carefully dissected after cardiac perfusion with 4% paraformaldehyde and PBS. To quantify Evans blue leakage, the spinal cord tissue from the injured region was homogenized in 50% trichloroacetic acid using a homogenizer (JXFSTPRP‐24L, Jingxin, China). After centrifugation, the Evans blue concentration in the supernatant was measured using a microplate reader (ELX808; BioTek, USA) at excitation and emission wavelengths of 620 and 680 nm, respectively.

### Statistical Analysis

All statistical evaluations were performed using Student's *t*‐test, Welch's *t‐*test, or one‐way analysis of variance (ANOVA). After ANOVA, the Bonferroni post‐hoc test or Dunnett's T3 post‐hoc test was used to adjust for multiple comparisons. Data are presented as mean ± standard error of the mean (SEM), and *p* values < 0.05 were considered statistically significant. Statistical analyses were performed using SPSS software (version 24.0, International Business Machines Corporation, USA) and GraphPad Prism (version 9.5.0, GraphPad Software, USA).

## Conflict of Interest

The authors declare no conflict of interest.

## Supporting information



Supporting Information

## Data Availability

The data that support the findings of this study are available from the corresponding author upon reasonable request.
