# Supplementary material for: AGAPIR: A Novel PIWI‐Interacting RNA Enhancing Post‐Decompression Angiogenesis in Degenerative Cervical Myelopathy
Source: Adv Sci (Weinh). 2025 Aug 18;12(42):e04246. doi: 10.1002/advs.202504246 (PMC12622476; doi:10.1002/advs.202504246)
Supplement: Supplementary file 1 — Supporting Information [file ADVS-12-e04246-s001.pdf]

WILEY-VCH

Supporting Information

AGAPIR: A Novel PIWI-Interacting RNA Enhancing Post-Decompression Angiogenesis in Degenerative Cervical Myelopathy

Yongheng Xie, Yiling Peng, Tianyu Qin, Cuimei Chen, Zhenxiao Ren, Naibo Feng, Chungeng Liu, Songlin Peng, and Houqing Long**

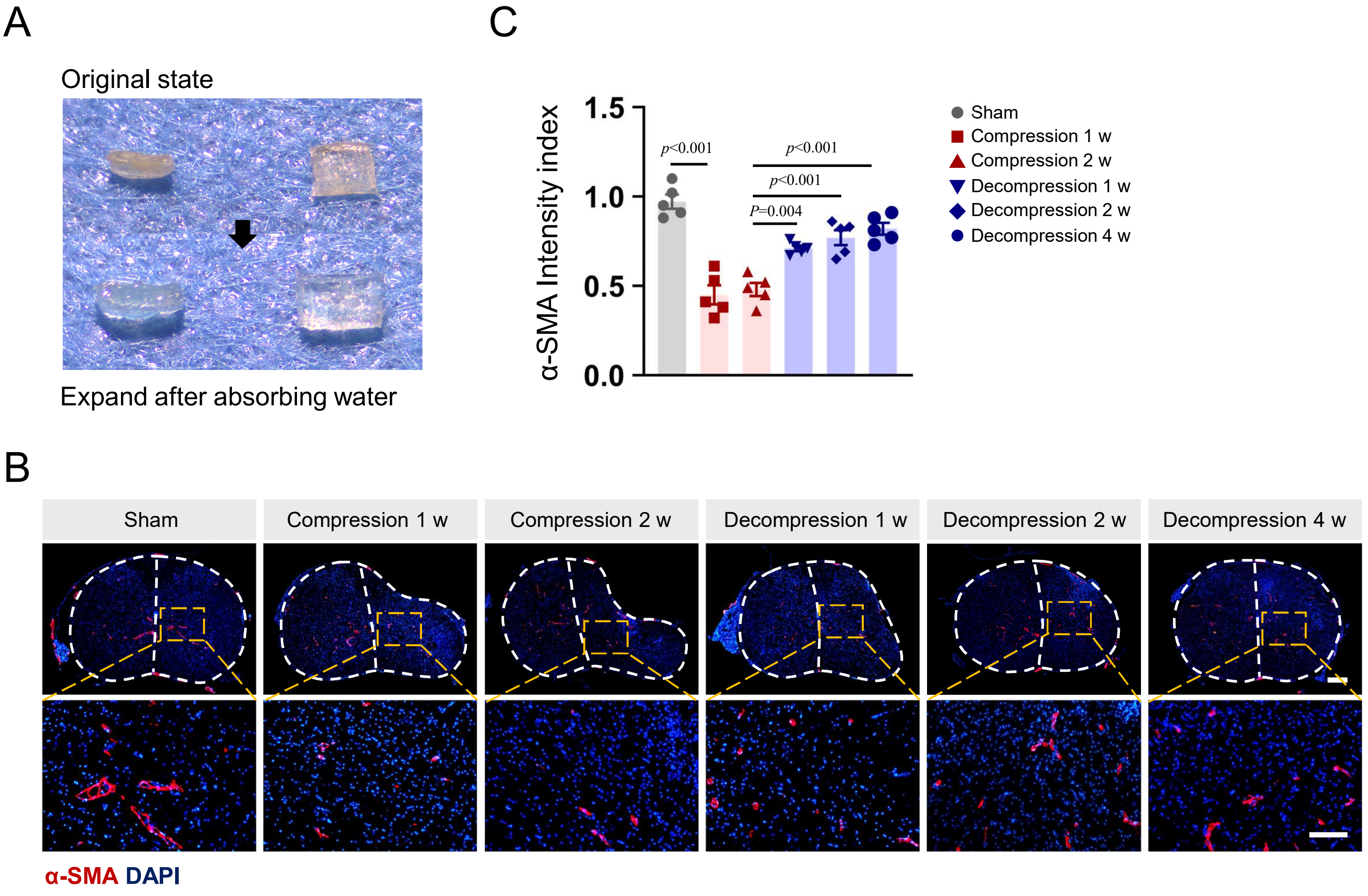

Figure S1. Surgical decompression promotes angiogenesis in the injured spinal cord. (A) Characterization of the hydrophilic polymer sheet demonstrating significant hydration capacity, with approximately 4-fold volumetric expansion upon water absorption compared to its dry state. (B) Representative immunofluorescence micrographs showing α-smooth muscle actin (α-SMA) expression patterns at the lesion epicenter, with (C) quantitative analysis revealing significant differences in mean fluorescence intensity among experimental groups. Data represent mean ± SEM (n=5 per group). Scale bars upper: 200 μm, lower: 100 μm. Statistical significance was determined by one-way ANOVA with Bonferroni's multiple comparison test.

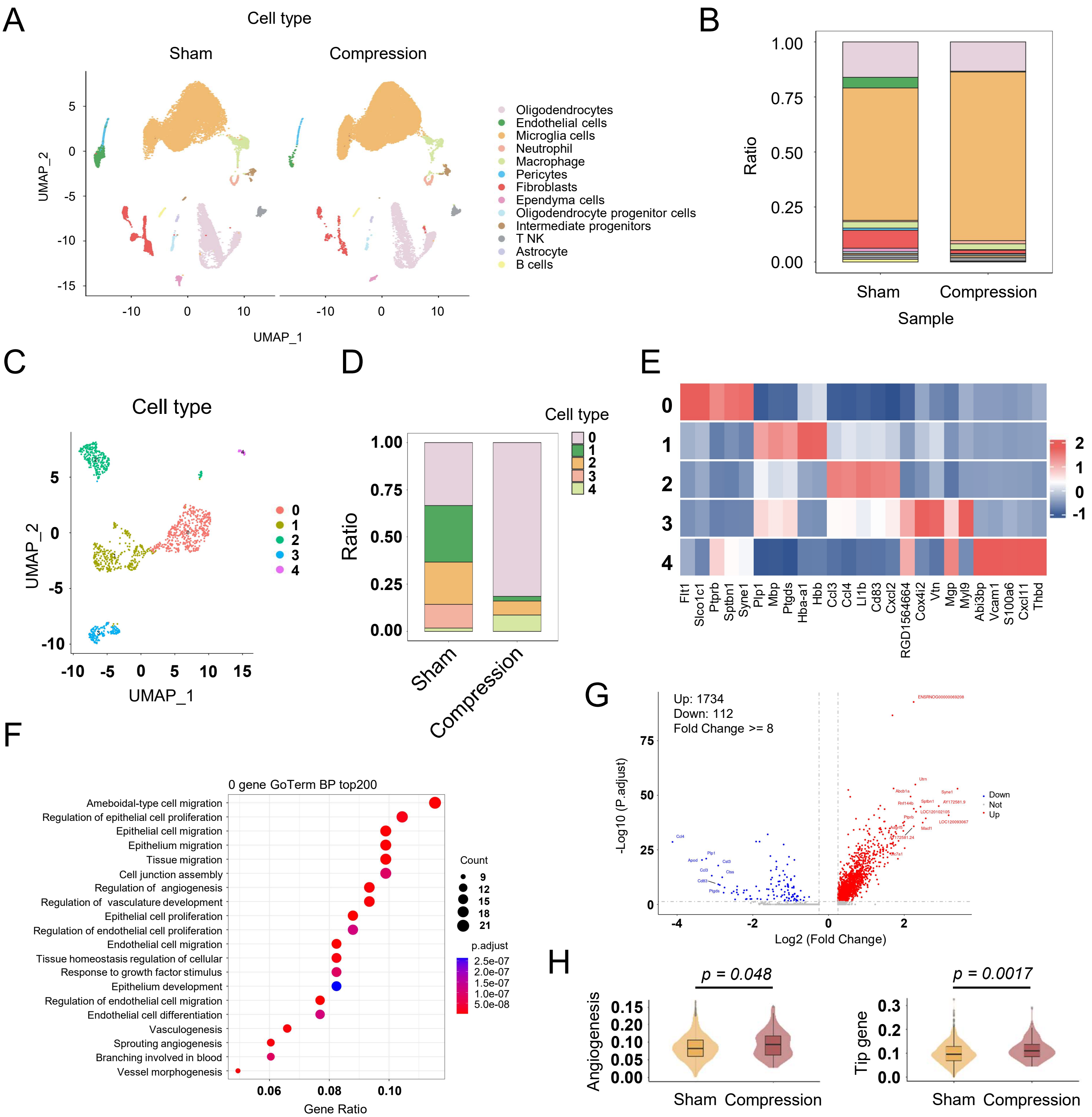

Figure S2. Single cell RNA sequencing data reveal transcriptomic features and changes in rat cervical spinal cord from sham and compression group. (A) Visualization of major classes of spinal cord cells by integrated UMAP plots of cell clusters. (B) Proportion analysis of cell subpopulations in sham and compression groups. (C) Endothelial cell subtypes visualized by UMAP clustering. (D) The proportion of each subpopulation of endothelial cells in the sham and compression group. (E) Heatmap of endothelial subtype-specific gene expression profiles. (F) GO analysis of cluster #0 showing enriched angiogenesis-related processes (regulation of endothelial proliferation/migration). (G) Volcano plot of differentially expressed genes between sham and compression groups. (H) Violin plots show the angiogenesis and tip gene expression in sham and compression group. Data from 3 rats per group. Statistical significance was determined by unpaired t-test.

A

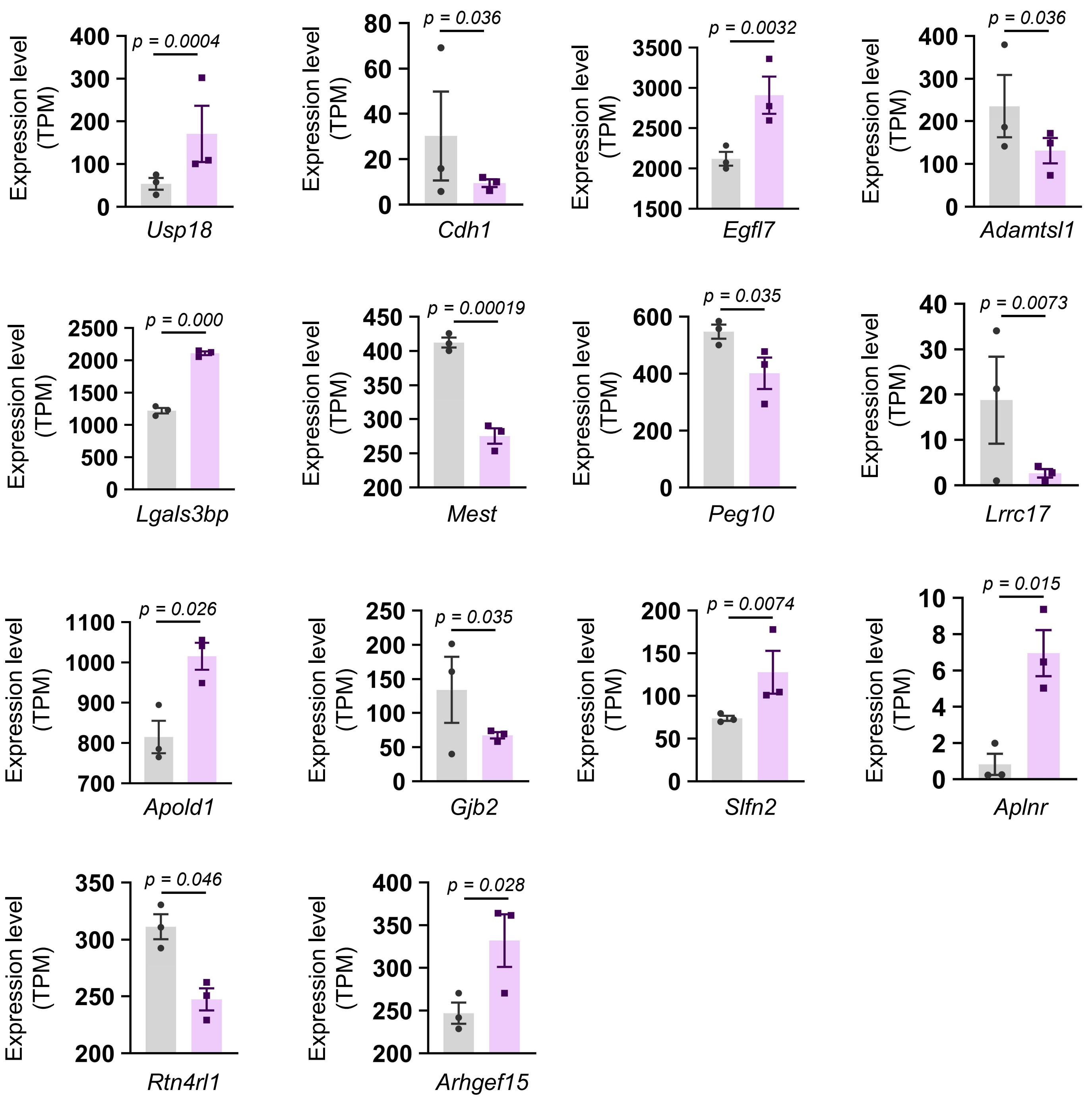

Figure S3. Transcriptome sequencing analysis revealed the expression profiles of genes predicted to interact with AGAPIR in spinal cord microvascular endothelial cells treated with either Negative control or AGAPIR agomir. Following cell collection and transcriptome sequencing, differentially expressed genes (DEGs) were identified. Concurrent bioinformatics analysis predicted candidate genes potentially capable of direct binding to AGAPIR, with their expression patterns subsequently compared between the Negative control and AGAPIR agomir-treated groups.

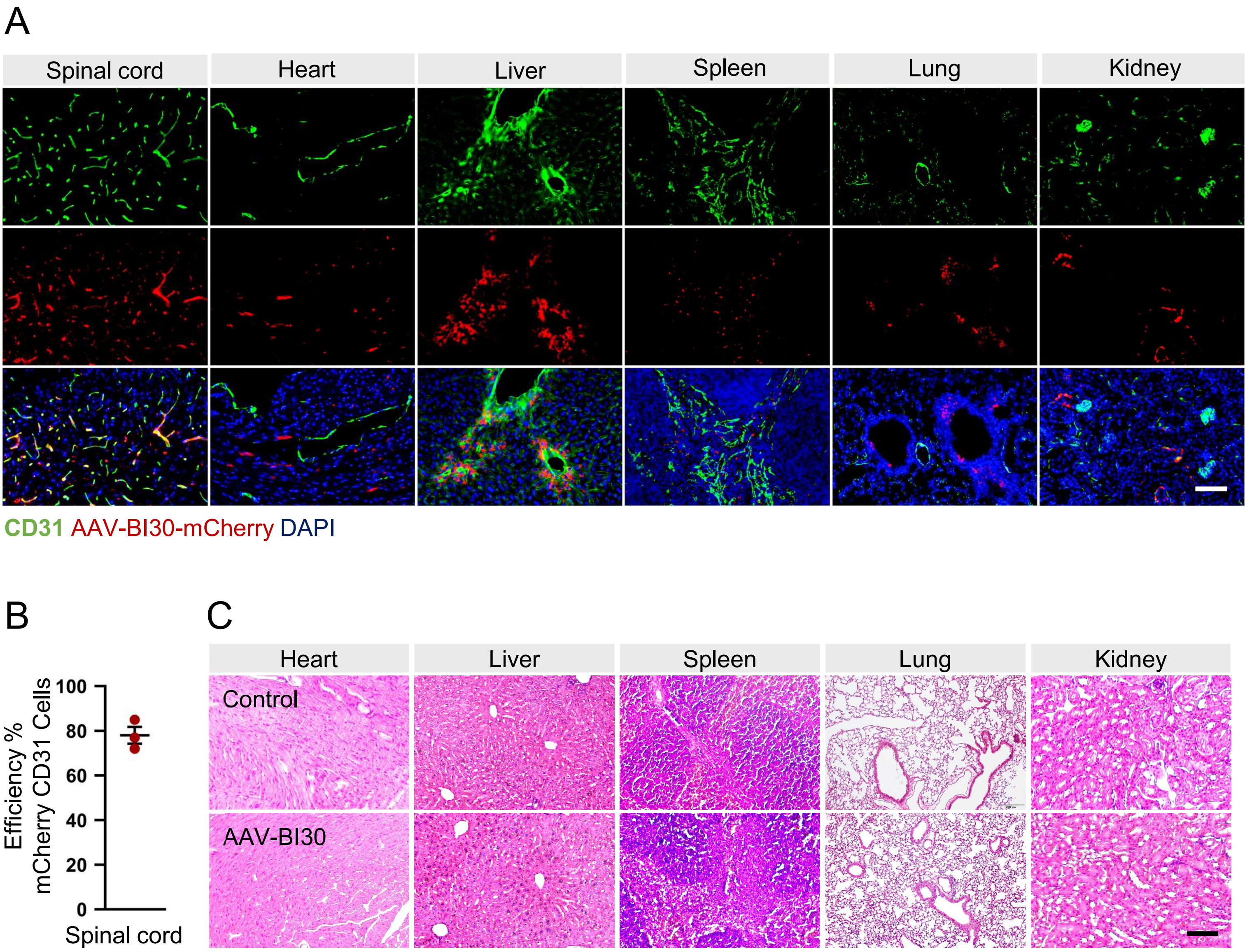

Figure S4. Systemic tropism characterization of AAV-BI30. (A) Viral biodistribution and endothelial targeting in 10-week-old C57BL/6 mice following intravenous injection (5×10^{11} vg/animal). Immunofluorescence at 4 weeks post-injection demonstrates AAV-BI30-mCherry transduction patterns (red) and colocalization with $CD31^{+}$ endothelial cells (green) across major organs, Scale bars: 100 μm. (B) Region-specific endothelial transduction efficiency was 78±7% in spinal cord. (C) Histopathological evaluation by H&E staining of heart, liver, spleen, lung, and kidney tissues from AAV-BI30-treated versus controls. Scale bars: 100 μm.

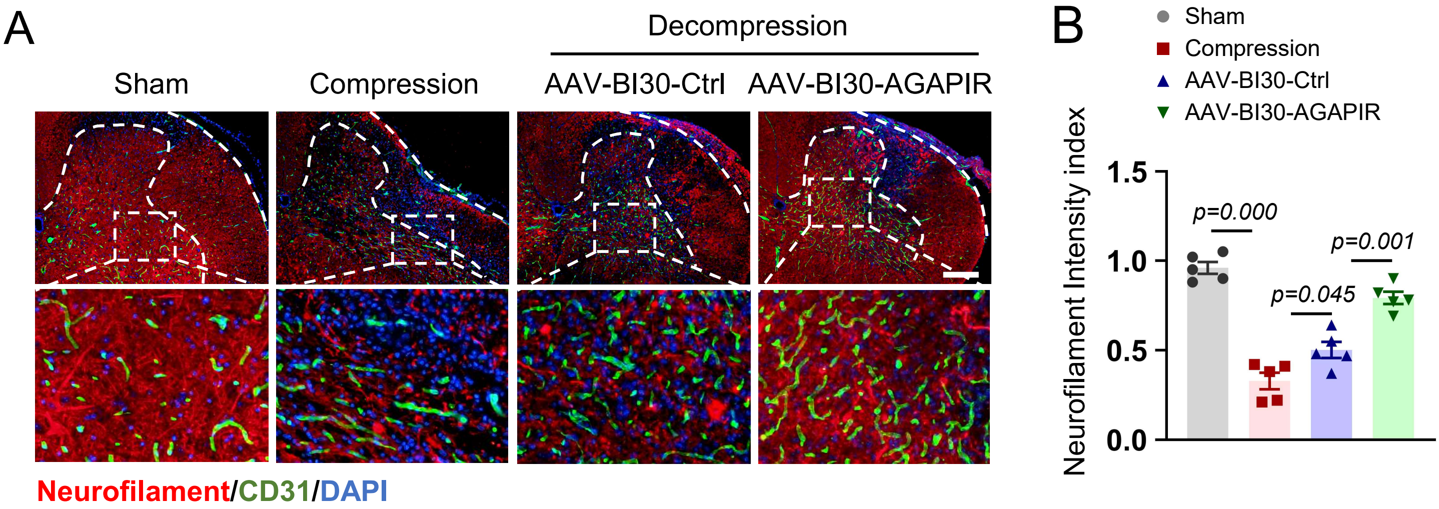

Figure S5. AGAPIR enhances neurofilament density recovery following spinal cord decompression. (A) Representative immunofluorescence images of neurofilament (red) and endothelial marker CD31 (green) expression in spinal cord tissues across experimental groups. (B) Quantitative assessment of neurofilament-positive fiber density. Scale bars: 200 μm. Data represent mean ± SEM (n=5 per group). Statistical significance was determined by one-way ANOVA with Bonferroni's multiple comparison test.

Supplementary Table S1. Primer Sheet

piRNAs	Seq 5'-3'
DQ540981	CGGGCCGCCGGTGAAATACCACTACTCTCA
DQ703900	GCGCCGCTGGTGTAGTGGTATCATGCAAGATT
DQ701563	TCCAGTGCGGTAACGCGACCGATCCC
DQ547181	TGAGAGGTGTAGAATAAGTGGGAGGCCC
uniq_2699	TGAGAACTGAATTCCATAGGCTGTT
uniq_2712	TGAGAACTGAATTCCATGGGTTAAA
uniq_2720	TCCCTGAGGAGCCCTTTGAGCCTGT
uniq_9221	TGCTCTACCGACTGAGCTATCCGGGC
uniq_9281	TAAAACCCATGGAATTCAGTTCTCA
uniq_9277	TTACAGCCTATGGAATTCAGTTCTCA
DQ541777	GGCTGGTCCGAAGGTAGTGAGTTATCTCAA
DQ719488	TTGGTCGCAGTTGAATGCTGTGTA
uniq_2671	TTCTCACTACTGCACTTGACTAGTCT
uniq_9194	GGATTAAGAGTCCCATGCTCTACCGA
uniq_2879	TCCCACTGCTTCACTTGACCAGCCTT
uniq_9387	AAGGCTGGTCAAGTGAAGCAGTGGGA
uniq_9543	TCAACAACCAGCTAAGACACTGCCA
uniq_3048	TGGCAGTGTCTTAGCTGGTTGTTGA
uniq_9321	TGGAGAGGATTTGAACCTCTGGGAAC
uniq_3676	CAGATCAGAAGGTGACTGTGGCTAT

Gene	Forward (5'-3')	Reverse (5'-3')
USP 18	TCTGTTCACCATCTGGACGC	CAAGGCATCCTCCAGGGTTT
HIF-1α	AGGATGAGTTCTGAACGTCGAAA	GGGGAAGTGGCAACTGATGA
Arhgef15	GGACTGTGAGTGTTCCCAGG	CAGGAAGCCCCTTAAGCCAA
Peg10	AAGGAGAGACGCCGCAAAAT	TCGCTGAAGGTCCCCCTATC
Rtn4rl1	ATGCTTCGCAAAGGGTGCT	GGATGGCAGCAAAGTTGTGT
Mest	CATCGAGTGATCGCCCTTGA	GCAGGTTGATTCTGCGGTTC
Gjb2	AGCTATGCATGTGGCCTACC	ACCACAGGGACCCTTCGATA
Egfl7	TGTCTACAGACCCAGCCGTA	CAGTCCGGTAGATGGTTCGG
Apold1	CATCGTGGGGTTGTCACTCA	CTCCCGGGAATTGCAGAAGA
Cdh1	AACCCAAGCACGTATCAGGG	ACTGCTGGTCAGGATCGTTG
Lrrc17	GGCCTCAAGATTTGCTGCAC	AAAGAACGCCTCGCTCTCAA
Aplnr	TCGTGGTGCTTGTAGTGACC	ATGCAGGTGCAGTACGGAAA
Lgals3bp	AGTTGCAGATCCCTGGGTTG	AGCTCTCCAGAGAGGTCCAG
Slfn2	GCAACTGAGCAAAGCAACCA	GCTCCGAGATTTAGACCCAGC
Adamtsl1	CTGCTCGTTCTGGCTTTCCTA	GCGTCTCAGGGAATAGGAGG
